# Supplementary material for: Translation elicits a growth rate‐dependent, genome‐wide, differential protein production in Bacillus subtilis
Source: Mol Syst Biol. 2016 May 18;12(5):870. doi: 10.15252/msb.20156608 (PMC5683663; doi:10.15252/msb.20156608)
Supplement: Supplementary file 1 — Appendix [file MSB-12-870-s001.docx]

**Appendix for**

**Translation elicits a growth-rate-dependent and genome-wide, differential production of proteins in *Bacillus subtilis***

Olivier BORKOWSKI^1,2^, Anne GOELZER^2^, Marc SCHAFFER^3^, Ulrike MÄDER^3^, Magali CALABRE^1^, Stéphane AYMERICH^1^, Matthieu JULES^‡1^ and Vincent FROMION^‡2^

*^1^ Micalis Institute, INRA, AgroParisTech, Université Paris-Saclay, 78350 Jouy-en-Josas, France; ^2^ MaIAGE, INRA, , Université Paris-Saclay, 78350 Jouy-en-Josas, France; and ^3^ Institute for Microbiology, Ernst-Moritz-Arndt-University Greifswald, 17487 Greifswald, Germany.*

^‡^ corresponding authors

Running title: Growth-rate-dependent bacterial translation

Table of content

1 Supplementary Methods 3

1.1 Growth conditions 3

1.2 The *fbaA* gene, a reference for constitutively expressed genes in *B. subtilis* 3

1.3 RNAs extraction and absolute quantification 3

1.4 Genome-wide DNA microarray 5

1.5 Real-time quantitative PCR 5

1.6 Culture of *B. subtilis* in a Live Cell Array 6

1.7 Estimates of growth rate and GFP abundance from LCA data 8

2 Supplementary Equations 10

2.1 A reduced model for protein production 10

2.2 The mathematical model of bacterial steady-state protein production for a given mRNA (m*_i_*) 11

2.3 Extension of the general model to the whole proteome 14

2.4 Alternative mathematical models 20

2.5 How do the Michaelis-Menten *versus* linear models of translation handle with growth-rate-dependent translation efficiency trajectories 26

3 Supplementary Notes 27

3.1 Estimate of ratios of protein production by “minimization of mean-square error” and bootstrap 27

3.2 Computation of the translation efficiency 28

3.3 The solution space of the K_1_, K_2_ and R*_free_* parameters 29

3.4 Genome-wide estimation of R*_free_* abundance and of the {K_1_*_i_*, K_2_*_i_*} pairs for each transcript 33

3.5 Higher R*_free_* abundance at slow than at fast growth should ensure that slowly growing cells are prone to adaptation 35

4 Supplementary Tables 36

4.1 Table S1: Protein productions in poor and rich growth conditions. 36

4.2 Table S2: Plasmids and Primers used in this work. 37

4.3 Table S3: Strains used in this work. 38

5 Supplementary Figures 39

5.1 Figure S1. The translation process is well described by a Michaelis-Menten like equation. 39

6 Supplementary references 40

# Supplementary Methods

## Growth conditions

Briefly, the S medium contained (NH4)_2_SO_4_ (0.2% w/v), K_2_HPO_4_ (1.4% w/v), KH_2_PO_4_ (0.6% w/v), sodium citrate (0.1% w/v), FeCl_3_/6H_2_O (0.001% w/v), Mg_2_SO_4_ (0.02% w/v), MnSO_4_ (0.056% w/v) and glucose (0.5% w/v). The TS medium is an S-based medium supplemented with L-glutamate (0.5% w/v) and Yeast extract (0.001% w/v). The CH medium (which contained 10% w/v casamino acid) was described in (Partridge & Errington, 1993). The CHG medium is a CH medium containing glucose (0.5% w/v). The modified M9 minimal medium (Harwood & Cutting, 1990) consisted of the following components (per liter): 8.5 g Na_2_HPO_4_•2H_2_0, 3 g KH_2_PO_4_, 1 g NH_4_Cl, 0.5 g NaCl. The following components were sterilized separately and then added (per liter of final medium): 1 ml 0.1 M CaCl_2_•2H_2_O, 1 ml 1 M MgSO_4_•7H2O, 1 ml 50 mM FeCl_3_•6H_2_O and 10 ml trace salts solution. The trace salts solution contained (per liter): 170 mg ZnCl_2_, 100 mg MnCl_2_•4H_2_O, 60.0 mg CoCl_2_•6H_2_O, 60.0 mg Na_2_MoO_4_•2H_2_O and 43.0 mg CuCl_2_•2H_2_O. Filter-sterilized carbon sources (pyruvate, succinate/glutamate, glucose or malate) were added separately to the medium at final concentration of 0.5% (w/v). Where necessary, carbon source solutions were pH neutralized with 4 M NaOH prior to addition to the medium.

## The *fbaA* gene, a reference for constitutively expressed genes in *B. subtilis*

By “genes under constitutive expression”, we meant genes for which the mRNA expression level solely depends on sigma factors and not on specific transcription factors. Several genes in *B. subtilis* can be considered as constitutive (Jules et al, 2009). In particular, the promoter of the *fbaA* gene was selected as a reference because it is well-known to be expressed independently from the major *B. subtilis* regulators (Goelzer et al, 2008; Ludwig et al; Tobisch et al, 1999) and to be under the only control of the housekeeping sigma factor (σ_A_) in exponentially growing *B. subtilis* cells (Nicolas et al, 2012).

## RNAs extraction and absolute quantification

### RNA isolation

For mRNA extraction, a single colony of *B. subtilis* was grown in 10 ml of LB medium during 2 hours. Then the samples, diluted 8000 fold in the medium of interest, were incubated with shaking at 37°C until they reached an OD_600_ of 0.1 to 0.5. Eventually, the samples were diluted 400 fold in the medium of interest and incubated in a 250 mL Erlen-meyer flask with shaking at 37°C until an OD_600_ of 0.5. Total RNA was isolated from cells using an acid phenol method. 30 ml samples were harvested by mixing with ½ volume of frozen killing buffer (20 mM Tris/HCl pH 7.5, 5 mM MgCl_2_, 20 mM NaN_3_) and subsequent centrifugation for 3 min at 8000 rpm at 4°C from exponentially growing cultures (OD_600_ 0.4 to 0.5). After discarding the supernatant, cell pellets were frozen in liquid nitrogen and stored at -80°C. For mechanical disruption, the pellets were resuspended in 200 μL of ice-cold killing buffer, immediately dropped into a Teflon disruption vessel (pre-cooled and filled in with liquid nitrogen) and then disrupted in a Mikro-Dismembrator S (Sartorius) for 2 min at 2600 rpm. The resulting frozen powder was resuspended in 4 mL of pre-warmed lysis solution (4 M guanidine thiocyanate, 25 mM sodium acetate pH 5.2, 5 g.l^-1^ N-laurylsarcosinate, 50°C) by repeated pipetting. Afterwards, 1 mL aliquots of the lysate were transferred to microcentrifuge tubes and immediately frozen in liquid nitrogen. Total RNA was isolated by acid-phenol extraction. Samples were treated twice with an equal volume of acid phenol/chloroform/isoamyl alcohol (25:24:1, pH 4.5, Carl Roth GmbH + Co. KG, Germany) and once with chloroform/ isoamyl alcohol (24:1, Carl Roth GmbH + Co. KG, Germany). After adding 1/10 volume of 3M sodium acetate (pH 5.2), RNA was precipitated with isopropanol, washed with 70% (v/v) ethanol and dissolved in 100 μL of RNase free water. 35 μg RNA were DNase-treated using the RNase-Free DNase Set (Qiagen) and purified using the RNA Clean-Up and Abundance Micro Kit (Norgen).

### RNA quantification

The RNA concentration and quality was checked using the Spectrophotometer Nanodrop ND-1000 (Thermo scientific, USA). The absorbance at wavelength of 260 nm in 1µl sample was used to measure RNA concentration. Absence of protein contamination in RNA extracts was checked measuring the absorbance at wavelength of 280 nm. Samples with 260 nm /280 nm ratios inferior to 2 were rejected. Absence of solvent contamination was verified with 230 nm /280 nm ratios and the low quality samples were rejected (ratio inferior to 2). The quality of the RNA preparations was assessed by means of the Agilent 2100 Bioanalyzer (Agilent Technologies, Palo Alto, CA, USA). The microfluidic glass chips (RNA 6000 Nano LabChip Kit, Agilent Technologies) were prepared according to the manufacturer’s instructions. Briefly, 1µl of sample diluted 10 fold and the RNA size ladder were incubated at 70 °C for 2 min, centrifuged at 16000 g for 10 s, and put on ice before running Bioanalyzer microchips. Agilent 2100 expert software was used for data analysis. The area of the 5S, 16S, 23S and precursors of rRNA peaks divided by the total RNA area gave the rRNA percentage and so the sum of the mRNA and tRNA percentages in the total RNA (**Figure EV1B-D**). The RNA 6000 Nano marker was used as an external standard for quantification. Interestingly, the pool of ribosomal RNA was enriched in precursors and depleted in 5S rRNA with increasing growth rates, suggesting that the rRNA maturation process is not efficient enough to compensate for the growth-related dilution of the implicated ribonucleases.

### RNA abundance as a function of the growth rate

Total RNA abundance at the different growth rates was fitted using a linear model. The plot shows that the residues are not homoscedastic. Such a behavior of residue variance is often observed when quantifying abundances. In order to obtain 95% confidence intervals on the regression parameters, and regarding the absence of homoscedasticity of the residues, we used the bootstrap procedure.

## Genome-wide DNA microarray

To 10 µg of each total RNA sample an equal amount of ten different in-vitro synthesized transcripts contained in the One Color RNA Spike-In kit (Agilent Technologies) were added. Afterwards, synthesis and hybridization of fluorescently labeled cDNA to Agilent custom microarrays were carried out as described previously (Winter et al, 2011), except that the individual samples were labeled with Cy3 and no common reference was used (one-color hybridizations). Data were extracted and processed using the Feature Extraction software (version 10.5, Agilent Technologies). Gene-level values were calculated from probe level data by error-weighted averaging using the Rosetta Resolver software (version 7.2, Rosetta Biosoftware). Finally, the median difference of the intensities of spike-in transcripts (considering only transcripts with a CV smaller than 25 %) was used to scale the gene-level intensities from each microarray in order to correct for technical variation associated with labeling and array processing. Processed data are provided in **Dataset EV1**.

## Real-time quantitative PCR

### Reverse transcriptase reaction

The reverse transcription protocol is composed of four steps. The first step was performed in 0.2 ml PCR tubes and corresponds to mixing in a volume of 11 µl, 1 µg of RNA and 0.25 µg of Pd(N)_6_. The mix was incubated at 70°C for 5 minutes. Then, 8 µl of the solution containing 1 µl of dNTPs 10mM (Invitrogen), 4µl of 5X Superscript buffer (Invitrogen), 2 µl of DTT 0.1M (Invitrogen) and 1µl of RNAse out 40U/µl was added and incubated 2 minutes at 42°C. Then, 1 µl of Superscript III 200U/µl (Invitrogen) was added and incubated 2 hours at 42°C. Eventually, the reaction was inactivated by incubation at 70°C for 10 minutes.

### Quantitative PCR reaction

We quantified cDNA in real-time qPCR using the SYBR green master mix kit (LightCycler^®^ FastStart DNA Master SYBR Green I, Roche) following the manufacturer’s recommendations. Briefly, each qPCR capillary contained 10 µl of reactions containing 0.5 µl of each primer 25mM, 1.2 µl of MgCl_2_ 25mM, 1µl of SYBR green master mix (Roche diagnostic), 4.3 µl of sterile water and 2.5 µl of cDNA solution. For each sample, two combinations of primers were designed using the LightCycler Probe Design Software 2.0 (Roche): (i) primers amplifying the reference *fbaA* mRNA, TGATCCTTGGTGTTTCCG / GTGAGAAGCGTCGATCATT (ii) primers amplifying the *gfpmut3* mRNA, ACATACGGAAAACTTACCCT / CATGGCACTCTTGAAAAAGTC. The corresponding DNA sequences were obtained from GenoList Database (http://genodb.pasteur.fr/cgi-bin/WebObjects/GenoList). The qPCR reactions were carried out in the LightCycler 1.5 instrument starting with a denaturing step of 10 minutes at 95°C and followed by 45 cycles (95°C, 10s; 56°C, 6s; 72°C, 10s). qPCR results are given relatively to the mRNA abundance of the reference *fbaA*).

### Quantitative PCR quality control

A combination of primers was also used to amplify the mRNA of the gene *pgi* CCTTCATTCTTTAGGCCAGTAT / GCAAGCATTGTACCTTGGA. The *pgi* mRNA abundance was compared to the *fbaA* mRNA abundance. The qPCR results together with microarrays analysis (BSB168 strain) allowed checking that the *^fbaA^*TIRs insertions didn’t disturb expression of *fbaA*. The sample contamination by DNA (due to a low efficiency of the DNAase before the step of reverse transcription) was checked by a qPCR on the RNA sample before performing the reverse transcription reaction. The samples with a RNA signal value superior to 2% (0.02-fold the expression of the gene of reference) were discarded.

### mRNA abundance as a function of the growth rate

We performed qPCR with 2 biological replicates and 4 technical replicates, which resulted into 8 values per strains and per medium. The qPCR datasets from *^fbaA^*TIRs and *^hs^*TIRs strains were fitted as function of the growth rate using a 2^nd^-degree polynomial and a 1^st^-degree polynomial, respectively. The 95% confidence bounds for the fitting coefficients are given by C=b+/-t$\sqrt{S}$. b is the vector of the coefficients of the fit; t is computed using the inverse of Student's t cumulative distribution function (MATLAB), and S is a vector of the diagonal elements from the estimated covariance matrix of the coefficient estimates, (X^T^X)^–1^s^2^. X is the design matrix and X^T^ is the transpose of X.s^2^ is the mean squared error.

Altogether "by combining total mRNA quantification, transcriptome data and qPCR data" we obtained quantitative expression profile of *gfp* across growth conditions for each synthetic strain.

To sum up at each µ: $\left[ {mRNA}_{gfpmut3} \right]=\left[ total RNA \right]*\alpha*\frac{\left[ {mRNA}_{fbaA} \right]}{\left[ total mRNA \right]}*\frac{\left[ {mRNA}_{gfpmut3} \right]}{\left[ {mRNA}_{fbaA} \right]}$

- $\left[ total RNA \right]$ was quantified by both Bioanalyser and Nanodrop
- $\alpha$ corresponds to the constant proportion of total mRNA abundance within total RNA as experimentally demonstrated using Spike-In control transcripts
- $\frac{\left[ {mRNA}_{fbaA} \right]}{\left[ total mRNA \right]}$ corresponds to the proportion of the *fbaA* transcript in total mRNA (mass) as quantified by genome-wide tiling arrays
- $\frac{\left[ {mRNA}_{gfpmut3} \right]}{\left[ {mRNA}_{fbaA} \right]}$ corresponds to the relative abundance of *gfpmut3* as compared to a control gene as quantified by qRT-PCR (in this case with *fbaA* as a control but was validated using *pgi* as another control)

## Culture of *B. subtilis* in a Live Cell Array

### Culture and data acquisition

For live cell array experiments, a single colony of *B. subtilis* was grown in a well of a 96-well microtiterplates (Cellstar^®^, Greiner bio-one) on LB medium until an OD_600_ of 0.4-0.5. For pre-cultures in the medium of interest, LB-grown cells were diluted 400-fold into microtiterplates and incubated under constant shaking at 37°C until OD_600_ reached 0.3. The cultures were initiated by diluting pre-cultures in 100 µl of the same medium to an OD_600_ of 0.001 and incubated at 37°C with constant shaking in a Synergy™ 2 multimode microtiterplate reader (BioTek^®^). OD_600_ and fluorescence (excitation 485/20 nm, emission 528/20 nm) were measured at an interval of 7 minutes (Botella et al, 2010; Buescher et al, 2012). Only the 60 wells located in the centre of the microtiter plates were inoculated, the wells around the edges exhibited evaporation higher than 5% over 20 hours and were only filled in with sterile medium. Each 96-well microtiterplate contained 12 replicates of the reference strain BSOB01 and of the four *gfp* reporter strains.

### Relationship between assessed fluorescence and intracellular GFP abundance

The measured fluorescence per well at any time (RawFluo) is related to the OD_600_-dependent GFP produced per well (GFP^OD^) but also reflects the OD_600_-dependent *B. subtilis* and medium auto-fluorescences (AutoFluo^OD^), the time-dependent intra-day variability (Drift*^t^*) and the day-to-day variability (D2D_Fluo*^d^*) as follows:

$$\mathbf{RawFluo}=\left( \mathbf{GFP}^{\mathbf{OD}}+\mathbf{AutoFluo}^{\mathbf{OD}} \right)\times\mathbf{Drift}^{\boldsymbol{t}}\times{\mathbf{D2D}\_\mathbf{Fluo}}^{\boldsymbol{d}}$$

In order to extract GFP^OD^ from the LCA data, we set up a specific design for the microtiterplate structure that sequentially allowed removing unwanted fluorescence.

#### Blank wells for the intra-day variability, Drift^t^

A drift of the measured fluorescence intensity was observed with time. A set of 6 external wells, only filled in by sterile medium, were used to estimate and compensate for the observed very slow drift of the excitation and receptor devices.

#### Fluorescein for the plate-to-plate normalization, D2D_Fluo^d^

In order to correct possible day-to-day variability between LCA experiments, we systematically included in each microtiterplates 6 wells filled in with different concentration of fluorescein (twice 10nM, 100nM and 1µM).

#### Dedicated wells for auto-fluorescence correction, AutoFluo^OD^

As already mentioned in the literature (Aichaoui et al, 2012; Botella et al, 2010), the raw fluorescence values are the sum of the GFP fluorescence level and the *B. subtilis* and medium-related auto-fluorescence (AutoFluo^OD^). We subtracted auto-fluorescences from the raw fluorescence values as previously described (Botella et al, 2010; Buescher et al, 2012). To this purpose, we systematically included in the 96-well microtiterplates, 12 replicates of the BSOB1 control strain.

#### Summary of a typical microtiterplate design

Following all these previous constraints, the 96-wells microtiterplate contained:

- 6 wells around the edges that were filled in by fluorescein (at the top left and bottom right of the microtiterplate)
- 6 wells on the right and left edges that were used as blank
- 60 wells that were inoculated by the different strains: 12 are for the BSOB1 control strain, and 12*4 for 4 *gfpmut3* reporter strains.

Due to the several unwanted fluorescences, the GFP abundance ratios between two strains were estimated within the same microtiterplate. 12 replicates for each strain allowed us to obtain a small 95% confidence interval for the estimated ratios.

### LCA experimental dataset

We performed 54 experiments (54 microtiterplates) as follows:

- M9P: 8 microtiterplates (4 with *^fbaA^*TIR strains and 4 with *^hs^*TIR strains)
- M9SE: 4 microtiterplates (2 with *^fbaA^*TIR strains and 2 with *^hs^*TIR strains)
- S: 7 microtiterplates (3 with *^fbaA^*TIR strains and 4 with *^hs^*TIR strains)
- M9G: 6 microtiterplates (3 with *^fbaA^*TIR strains and 3 with *^hs^*TIR strains)
- M9M: 4 microtiterplates (2 with *^fbaA^*TIR strains and 2 with *^hs^*TIR strains)
- TS: 6 microtiterplates (2 with *^fbaA^*TIR strains and 4 with *^hs^*TIR strains)
- CH: 9 microtiterplates (6 with *^fbaA^*TIR strains and 3 with *^hs^*TIR strains)
- CHG: 10 microtiterplates (7 with *^fbaA^*TIR strains and 3 with *^hs^*TIR strains)

Raw data are available in supplementary excel files.

## Estimates of growth rate and GFP abundance from LCA data

### Definition of the exponential steady-state regimen in LCA

After systematic removal of non-growing cultures, we determined the “OD_600_ range” in which all clones were considered to be in exponential growth and therefore in steady-state regimen. The range was manually defined based on the best linear fit of the OD_600_ (in logarithmic scale) with respect to the time. This step allowed further removal of data from specific wells in which cells did not grow in steady-state regimen.

### Growth rate calculation

In order to estimate both the growth rate and the GFP abundance (GFP per well divided by OD at each time point), we subtracted in each well the first measured value of OD_600_ (time 0) to all OD_600_ measurements. The corrected OD_600_ time-series in logarithmic scale were fitted by a linear model, which directly provided the corresponding growth rates (its slopes). Then, the median of the 12 growth rates obtained per strain in each microtiterplate was used as the reference growth rate per strain and per experiment. The 95% confidence intervals for the different parameters were obtained by bootstrap (Efron, 1987).

### Estimate of GFP abundance at different growth rates

#### Intra-day compensation, Drift^t^

We estimated the drift using a loess regression as function of time, and we obtained the desired compensation after a suitable normalization, $\alpha_{0}$ which is such that

$$\alpha_{0}\int_{0}^{\mathrm{tf}} \mathrm{Drift}\left( t \right)\mathrm{dt}=1$$

Eventually, RawFluo data were corrected at each time point as follows:

$$RawFluo\_corrected \left( t_{i} \right)=\frac{\mathrm{RawFluo}\left( t_{i} \right)}{\alpha_{0}\mathrm{Drift}\left( t_{i} \right)}$$

***Nota Bene:*** in the sequel, we will use the term “RawFluo” in place of “RawFluo_corrected”.

#### Normalization by fluorescein, D2D_Fluo^d^

Fluorescein is a light-sensitive molecule that is subject to photobleaching. Therefore, we normalized fluorescence levels of the entire dataset using the mean value of the fluorescein-related fluorescence obtained during the only 20 first measurements (for a total interval of 140 minutes).

#### Auto-fluorescence correction

In order to estimate GFP fluorescence, we subtracted an auto-fluorescence function from the raw fluorescence datasets. We defined the auto-fluorescence function using a 3^rd^-degree polynomial applied to the OD_600_ and fluorescence measurements of the control strain (BSOB1):

AutoFluo^OD^(OD) = b_0_ +b_1_ OD+ b_2_ OD^2^ +b_3_ OD^3^

where OD is used in place of OD_600_

The GFP abundance during the exponential phase is calculated for each time point (t_i_) as follows:

$$[GFP (t_{i})]=\frac{RawFluo (t_{i}) - \mathrm{AutoFluo}^{\mathrm{OD}}(OD(t_{i}))}{OD(t_{i})}$$

The final GFP abundance is given by the median of $\left[ \mathrm{GFP}\left( t_{i} \right) \right]$ with time.

Processed data are provided in **Dataset EV1**.

# Supplementary Equations

The aim of this section is (*i-*) to provide an overview of the molecular entities of the reduced model of protein production depicted on **Figure 7** (the reduced model is called in the main text as the “synthetic model” or "complete model"); (*ii-*) to derive from these molecular entities the mathematical model used in this study; (*iii-*) to explain and investigate the consequences of some assumptions made while building the mathematical model.

It is well-established that the translation initiation can be divided into numerous elementary reaction steps that are reversible and irreversible (Antoun et al, 2006). Modeling the whole initiation process as described in (Milon et al, 2012) is mathematically achievable but leads, even in steady state, to a complex, nonlinear model that depends on a large number of species abundances and rate constants. Reducing the complexity of the complete steady-state nonlinear model was a key issue that droves our developments. To this purpose, we focused on the Michaelis-Menten like contribution of several molecular entities in the complete steady-state nonlinear model. To reduce the complexity of the complete steady-state nonlinear model, we verified for each molecular step the available biological knowledge to determine if the Michaelis-Menten function could be consistently reduced. However, the magnitude of the reduction always obeys to a subtle trade-off between model “simplicity” and model “capability” to accurately catch the biological, experimental knowledge. Eventually, our reduced steady-state model is simple as compared to the complete steady-state nonlinear model but still catches the essence of our biological observations.

## A reduced model for protein production

We divided the translation process into 3 initiation steps (from the binding to the initiation) and a completion step (elongation and termination) (Milon et al, 2008; Ramakrishnan, 2002). Protein abundance was obtained by additionally integrating protein degradation and protein dilution (**Figure 7**, **Table 2**). Translation initiation is divided into two reversible steps, (a) ribosome binding onto the mRNA and (b) accommodation of the ribosome onto the start codon, which are followed by one irreversible step, the initiation of translation elongation.

Ribosome binding to the mRNA (**Step 1**) is a reversible step (Antoun et al, 2006). The k*_bi_* and k*_-bi_* affinity constants correspond to the probability of encounter/dissociation of R*_free_* with/from the ribosome-docking site (RDS) of a given mRNA (m*_i_*, (Antoun et al, 2006)). These constants depend on the ribosome binding site (RBS) sequence (within the RDS) and on the structural availability/occupancy of the RDS (Salis et al, 2009; Schubert et al, 2007; Vellanoweth & Rabinowitz, 1992). Accommodation of the ribosome to the start codon (**Step 2**) is also a reversible step (Antoun et al, 2006). It reflects the ability of the 30S subunit to set on the start codon. The k*_ai_* and k*_-ai_* affinity constants primarily depend on the distance between the RBS and the start codon and possibly on the neighboring nucleotides (Antoun et al, 2006). Initiation of translation elongation (**Step 3**) is an irreversible step (for a review see (Ramakrishnan, 2002)). The 30S🞄mRNA🞄IFs🞄GTP🞄fMet-tRNA^fMet^ ribosomal complex undergoes a large-scale conformational change promoted by the hydrolysis of a GTP molecule by IF2 which allows the recruitment of the 50S subunit (Milon et al, 2012; Tomsic et al, 2000). Then, the 70S ribosome is ready to catalyze the first peptide bond of the nascent protein (Tomsic et al, 2000). Translation elongation is also an irreversible step (Ramakrishnan, 2002) proceeding at a growth-rate-dependent rate, k*_pi_*. In the reduced model displayed on **Figure 7**, as in a complete steady-state nonlinear model integrating each reaction step as described in (Antoun et al, 2006), initiation of translation is the limiting step of protein production for most of the mRNAs in steady-state growth. This is consistent with the observed higher ribosome density at the beginning of coding sequences (vicinity of the Shine-Dalgarno sequence) for the ~2000 most highly expressed genes in *E. coli*, which reflects the slower kinetic of translation initiation (Oh et al, 2011).

One simplification of the reduced/general model concerns the role of the *free* 50S subunit in steady-state growth. Indeed, in the presence of IF1 and IF3, the *in vitro* rate of formation of the 70S initiation complex is weakly dependent on the abundance of the *free* 50S subunit when the *free* 50S subunit abundance is high enough (Antoun et al, 2006). To strengthen this assumption, we note that *in vivo*, both the genetic organization of ribosomal genes in *B. subtilis* (Kunst et al, 1997) and the existence of an autogenous translation feedback of ribosomal proteins that control ribosome stoichiometry suppose an *approx.* equal abundance of 30S and 50S subunits (Grundy & Henkin, 1991; Kaczanowska & Ryden-Aulin, 2007). Since the *free* 30S subunit is distributed within several untranslating ribosomal complexes (**Figure 7, Table 2**), for modeling ribosome assembly we assumed that the *free* 50S subunit is more abundant than the 30S🞄mRNA🞄IFs🞄fMet-tRNA^fMet^ complex. This assumption leads to a model, where the initiation process is summarized by two reduced steps: a first reversible step which corresponds to the ribosome accommodation onto the start codon, followed by an irreversible step, which corresponds to the initiation of translation elongation.

## The mathematical model of bacterial steady-state protein production for a given mRNA (m*_i_*)

In this section, we derived the system of differential equations associated to the model described on **Table 2**. We then computed the nonlinear model associated to the protein production in steady-state growth. Let’s first introduce some notations.

**We defined 4 sub-forms of ribosomal species:**

1. *Free* (untranslating) ribosome (R*_free_*, 30S🞄IFs🞄fMet-tRNA^fMet^), which corresponds to the ribosomes that are available for all mRNAs.
2. Ribosome bound to a given mRNA (m*_i_*), which corresponds to the ribosomes that form a complex (R_b_*_i_*) that impedes the access of other ribosomes to the translation initiation region (TIR).
3. Ribosome accommodated onto the mRNA (m*_i_*) start codon, which corresponds to the ribosomes that form a molecular complex (R_a_*_i_*) that still impedes the access of other ribosomes to the translation initiation region (TIR). Indeed, when bound to an mRNA, a ribosome covers around 30 nucleotides (Steitz, 1969) and about 90% of the RBS in *B. subtilis* are distributed between positions -5 and -11 relatively to the start codon (Rocha et al, 1999).
4. Ribosome initiating translation elongation (R_t_*_i_*) corresponds to the ribosomes that release the TIRs, which therefore become free for binding another ribosome (R*_free_*).

**We introduced a sub-form of mRNA:**

*Free* m_i_ (m*_fi_*) that expose their TIR to ribosome recruitment (we note that the two other messenger species have already been described as a part of R_b_*_i_* and R_a_*_i_* complex).

We formalized the molecular steps described above and obtained the following differential equations relating ribosome and m*_i_* abundances:

|  | (**1**) |
| --- | --- |
|  | (**2**) |
|  | (**3**) |
|  | (**4**) |

And the conservation of the mass for mRNA of a given gene *i*:

|  | (**5**) |
| --- | --- |

***Comments:***

1. We have assumed that the R_a_*_i_*, R_b_*_i_* and R_t_*_i_* ribosomal and m*_fi_* species are all involved in fast chemical reactions so that their respective dilution rate can be assumed to be negligible as compared to the rates of transition from one species to another.
2. We have assumed that *free* 50S abundance is high enough so that the rate of “initiation of translation elongation” (**Step ➂**) is saturated and then does not depend on the *free* 50S abundance. Thus, in the reduced/general model we wrote “ ” and not “  ”.

1. We have assumed that protein degradation is negligible as compared to dilution, *i.e.* γ_i_ << *µ*. In the exponential phase of growth, most of the cytoplasmic proteins of *B. subtilis*, and more generally of bacteria, are highly stable (Gur et al, 2011; Jayapal et al, 2010; Kock et al, 2004; Piir et al, 2011). Nevertheless, if this assumption was not satisfied for a given protein, we would need to replace “*µ*” by “*µ* + γ_i_”.

In steady-state growth, **equations** (1) to (4) can be rewritten as

| \|  \| (**6**) \| \| --- \| --- \| \|  \| (**7**) \| \|  \| (**8**) \| \|  \| (**9**) \| |  |
| --- | --- | --- | --- | --- | --- | --- | --- | --- | --- |

In order to have a closed-form expression of the protein abundance, we expressed the abundance of each ribosomal entity as a function of R*_free_*.

**Equation** (5) and **equation** (6) lead to the following expression

| .  | (**10**) |
| --- | --- |

By combining **equations** (7) and **equations** (**10**), we have

| .  | (**11**) |
| --- | --- |

**Equation (8)**, *i.e.*, irreversibility of the initiation of translation elongation, leads to:

|  | (**12**) |
| --- | --- |

We consequently have the protein production as a function of , *i.e.*,

|  | (**13**) |
| --- | --- |

The combination of **equations** (**11)** and **equation** (**13)** directly gives the relationship between protein and *free* ribosome abundances:

|  | (**14**) |
| --- | --- |

Eventually, we introduced a more compact expression of **equation** (**14)**, which we called the “**synthetic model**” in the main text**:**

|  | (**15**) |
| --- | --- |

where and

***Comment:*** The **equation** (**15)** highlights why it is not necessary to detail the elongation step since protein abundance does not depend on it. This is a direct consequence of the irreversibility of **step ➂** (initiation of translation elongation), which obviously implies that there is “no traffic jam of ribosomes” that may prevent a new cycle of translation initiation in steady-state growth.

## Extension of the general model to the whole proteome

Using the reduced/general model of bacterial protein production, we extended our analysis to the whole proteome. In this analysis, we assumed that protein degradation is negligible since ribosomal and cytosolic proteins were found to be stable during exponential growth in Gram-negative and Gram-positive bacteria (Gur et al, 2011; Jayapal et al, 2010; Kock et al, 2004; Piir et al, 2011).

### Mathematical framework

To extend the analysis to the whole proteome, we introduced a class of genes regulated at the translational level (even if only a few genes are known to be regulated at the translational level, (Kaczanowska & Ryden-Aulin, 2007)). Two classes of regulatory mechanisms were considered: (➊) regulatory mechanisms preventing the binding of *free* ribosomes, (➋) regulatory mechanisms interfering with another step of translation initiation.

Regulatory mechanisms preventing the binding of *free* ribosomes (➊) lead to the following model:

,

where corresponds to the level of regulation of the messenger *i*. is less than or equal to 1. To this purpose, we assumed that a part of the *free* messenger, *i.e.* is now sequestered in an inactive form (not able to bind a *free* ribosome) denoted in the sequel. A new differential equation is now integrated in the model:

where is the rate of “inactivation” of a *free* messenger and is the rate of messenger “activation”. In steady-state regimen, we then have . The inactive form of the messenger is also part of the mass conservation equation (**5)**, which now can be rewritten as: .

Therefore, we have: . By introducing, we obtain:

,

which leads to:

where and are the same as in equation (**15)**. The case of the regulatory mechanism (➊) then results in steady-state growth condition to an apparent ’ parameter, which corresponds to the real increased by .

**The case of regulatory mechanisms interfering with the translation initiation but independently from the *free* ribosome binding** (➋) led us to evaluate several cases since the mathematical derivation depends on the molecular step being targeted by the considered translational mechanism. These regulatory mechanisms also lead to ‘apparent’ and parameters. For the sake of simplicity, we assumed in the sequel that the messengers regulated at the translation level are all regulated as in case (➊) since handling the case (➋) is not more difficult to derive and does not modify our general conclusions.

### Extension to the whole proteome

Total ribosome abundance, *i.e.* [R_Tot_], within a cell corresponds to the sum of all individual ribosomal species, *free* or bound to any unregulated m*_i_* (from 1 to *n* genes) or any regulated m*_i_* (from 1 to m genes):

|  | (**16**) |
| --- | --- |

Following previous mathematical derivations the abundance of each ribosomal entity associated to unregulated genes can be expressed as a function of [R*_free_*]: , and . For regulated genes, we have: , and . Consequently, replacing each ribosomal entity in **equation** (**16)** we obtain:

|  | (**17**) |
| --- | --- |

and then

|  | (**18**) |
| --- | --- |

where K_1_*_i_* and K_2_*_i_* constants are identical to those from the reduced/general model (see **equation** (**15**)).

The previous equation can be rewritten in a more compact form:

|  | (**19**) |
| --- | --- |

with $\text{K}_{2i}=\text{K}_{2i}$ for $i\in(1,...,n)$ and $\text{K}_{2(i+n)}=\text{K}_{2i}/\beta_{ri}$for $i\in(1,...,m)$.

The qualitative analysis of the steady-state abundance of *free* ribosome to variations of total mRNA and ribosome abundances is shown on **Figure 7D** and described in the main text.

**Remark**. The first term of the right hand side of equation ((**18)**) is a weighted sum of messenger abundances where the weight attached to gene *i* is given by:

This weight is decreasing with respect to the growth rate since (i) [R*_free_*] is a decreasing function of the growth rate and (ii) $\text{k}_{\text{p}i}$ is increasing with growth rate since the translation speed increases with growth rate (Bremer & Dennis, 2008).

The same remark occurs for the weights associated to the second term of the right hand side of equation ((**18)**) and given by

However, $\beta_{ri}$ depends on the considered condition and its effects on the weight value is then unknown.

### Putative effect of the autogenously regulated translation of ribosomal proteins on R_free_ abundance

The autogenous regulation of ribosomal protein translation falls into the previously formalized case ➊ (**§2.3.2**). We therefore investigate if the behavior of R*_free_* abundance with respect to the growth rate could be explained by the increasing weight of ribosomal proteins within the whole proteome as a function of the growth rate.

The stoichiometry of proteins in the 30S and 50S complexes implies that the abundance of each ribosomal protein is almost a multiple of $[\text{R}_{\text{Tot}}]$. For $N_{r}$ distinct ribosomal proteins present in the ribosome for a considered condition, the abundance of the ribosomal protein ‘r*_i_*‘ with $i\in(1,...,N_{r})$ is by definition where ‘$\alpha_{i}$’ corresponds to its stoichiometry in the ribosomal complex (because in view of the highly elevated ribosome abundance, we assumed that abundances of ribosomal proteins associated to ribosomes under maturation and of *free* cytosolic ribosomal proteins can be neglected).

Regardless the regulatory mechanisms controlling the production of ribosomal proteins, the abundance of each ribosomal protein can be described as

If no regulatory mechanism exists, . Otherwise, .

We then deduce for the whole ribosomal proteome that:

and then for the whole proteome:

which simplifies into:

with $\text{K}_{2i}=\text{K}_{2i}$ for $i\in(1,...,n)$ and $\text{K}_{2(i+n)}=\text{K}_{2i}/\beta_{ri}$for $i\in(N_{r}+1,...,m)$.

This expression is close to that of equation ((**18)**), therefore allowing similar qualitative analysis. As long as remains an increasing function of the growth rate, the conclusions presented in the main text during the analysis of expression ((**18)**) hold true. Under this assumption, we conclude that the behavior of [R*_free_*] is not fully imposed by the behavior of the ribosomal proteins with respect to the growth rate. Eventually, there exist non-ribosomal proteins that also contribute to decrease the abundance of R*_free_* with increasing growth rate.

The behavior of can be further investigated based on data from *E. coli*(Marr, 1991) and here we show that remains an increasing function for *E. coli.*

with

The right hand side of the previous expression corresponds to the proportion of ribosome used for producing each considered category. That means for example that the second terms represents from 10% to 25% of the whole protein production when growth rate belongs to 0.5 to 2 in view of the data from *E. coli* (Marr, 1991).

For growth rates belonging to [0.5, 2], we have

and then

Actually, since , we then have more surely

The total quantity of ribosome is increasing linearly with the growth rate for growth rate belonging to [0.5, 2]:

$$\text{[R}_{\text{Tot}}\text{] = }a\text{ }+\text{ }b\text{ }\mu$$

Following the previous derivation, we then have

and then

The left hand side of the previous equality is an increasing function of the growth rate if:

$$\mu\leq\frac{b-bc-da}{2db}=\frac{1-c-d\frac{a}{b}}{2d}$$

We have to estimate *c*, *d* and *a/b.* Following the data from *E. coli* (Marr, 1991), we have:

$$\left\{\begin{matrix} \text{ }a\text{ }+\text{ }0.5\text{ }b\text{ }= 30 \text{for} \mu=0.5 \\ a\text{ }+\text{ } 2 b\text{ }= 60 \text{for} \mu=2 \end{matrix}\right.$$

and then *b*=20 and *a*=20 thus *a/b*=1. For *c* and *d*, we have

$$\left\{\begin{matrix} c+0.5 d= 0.10 \text{for} \mu=0.5 \\ c+2 d = 0.25 \text{for} \mu=2 \end{matrix}\right.$$

and thus *d*=0.1 and *c*=0.05.

We finally conclude that $(a+b\mu)\left(1-c-d \mu\right)$ is an increasing function of growth rate if

$$\mu\leq\frac{1-0.05-0.1}{0.2}=4.25$$

and then is an increasing function for growth rate belonging to [0.5,2] (if the simplification derived by assuming is not used, we then have shown that is greater than this quantities $\left(a+b\mu\right)\left(1-c-d\mu\right)$ and then greater than a function which is strictly increasing function of the growth rate. Altogether, it suggests that the synthesis of ribosomal proteins cannot alone explain the drop of R*_free_* abundance.

## Alternative mathematical models

Translation efficiency in the form of a Michaelis-Menten-like equation ensued from straightforward, reasonable biological assumptions. As for the model in which translation efficiency was considered as constant (Klumpp et al, 2009), one can be surprised that translation efficiency in the form of a rather simple Michaelis-Menten-like equation can properly illustrate a sophisticated process like translation. The general model considered 4 major steps for protein production but a dozen of molecular steps have already been described (Milon et al, 2012; Ramakrishnan, 2002). We also considered that *free* 50S abundance is high enough so that the rate of “initiation of translation elongation” does not depend on the *free* 50S abundance. We therefore explored more complex models to verify to which extend translation efficiency can simplify into a Michaelis-Menten-like equation and under which assumptions, but also to gain insights on what lies behind the newly defined global, growth-rate-dependent R*_free_* variable. Thanks to the synthetic model, we deduced both the relative variation of R*_free_* abundance and the values of the K_1_*_i_* and K_2_*_i_* aggregated constants that originate from the most probable *in vivo* ribosome assembly based on *in vitro* data. However, it was shown *in vitro* that mRNAs can also bind to the ribosome at any time before or after the binding of the initiation factors (IFs) and the initiator fMet-tRNA^fMet^ (Milon et al, 2012). Due to the intrinsic nature of ribosome assembly, it is likely that there are *in vivo* several ways of building the initiation complex and initiating translation. We therefore developed additional models, which provide some insights on the biological process of protein production and whose conclusions drove the reduction of the complete steady-state nonlinear model to the reduced/general model. We had considered alternative models integrating explicitly the role of:

*a.* the *free* 50S abundance,

*b.* alternate orders of binding of the IFs and tRNA^fMet^ onto the 30S subunit

### A model integrating the free 50S abundance

The additional model includes one new (**step 2.2**) and one modified (**step 3**) molecular steps right after ribosome accommodation (usual **step 2**), which corresponds to the recruitment of the 50S subunit (**step 2.2**) and to the initiation of translation elongation (**step 3**), respectively. As compared to the general model, the specific properties of the additional model are summarized below:

| **Step 2.2** | ***50S docking***  | R_a_*_i_*=30S•IF1•IF2•IF3•tRNA^fMet^•m*_i_* (pre-initiating ribosome)  R_s_*_i_*=50S•30S•IF1•IF2•IF3•tRNA^fMet^•m*_i_* (docking ribosome)  50S*_free_*= *free* 50S subunit of the ribosome  k_s_*_i_*= rate of *free* 50S docking to R_a_*_i_*  k*_-_*_s_*_i_*= rate of *free* 50S release from R_s_*_i_* |
| --- | --- | --- |
| **Step 3** | ***Initiation of translation elongation***  | R_t_*_i_*= 50S•30S•IF1•IF2•IF3•tRNA^fMet^•m*_i_* (initiating ribosome)  k_t_*_i_*= rate of initiation of translation elongation |

The differential equations related to the molecular system described above are:

|  | (**20**) |
| --- | --- |
|  | (**21**) |
|  | (**22**) |
|  | (**23**) |
|  | (**24**) |

We can write the new conservation of mass:

|  | (**25**) |
| --- | --- |

The steady-state growth model is then the solution of this set of equations:

|  | (**26**) |
| --- | --- |
|  | (**27**) |
|  | (**28**) |
|  | (**29**) |
|  | (**30**) |

**Equation** (**29)** and **Equation** (**30)** lead to:

|  | (**31**) |
| --- | --- |

We now expressed the ribosomal quantities as a function of [R*_si_*],

From **Equation** (**28)** , we have:

| . | (**32**) |
| --- | --- |

**Equation** (**27)** and **Equation** (**32)** lead to

| . | (**33**) |
| --- | --- |

In order to express the *free* messenger abundance as a function of [Rsi], we use **equation** (**26)**:

|  | (**34**) |
| --- | --- |

and obtained the announced result using **equation** (**25)**:

|  | (**35**) |
| --- | --- |

To conclude, we combined **equation** (**31)**, **equation** (**34)** and **equation** (**35)**:

|  | (**36**) |
| --- | --- |

which can be rewritten as

|  | (**37**) |
| --- | --- |

with

and

K’_2,50S_*_i_* can be written as a function of K’_1,50S_*_i_*:

The *free* 50S abundance appears in the nonlinear expressions of the two pseudo-coefficients, K’_1,50S_*_i_* and K’_2,50S_*_i_*. Regarding our experimental results, the two pseudo-coefficients must be near constant. Two scenarios can be envisaged:

1. The *free* 50S abundance is invariant with respect to the growth rate. The well-known variations of total ribosome abundance with growth rate (Bremer & Dennis, 2008; Schaechter et al, 1958) would then suggest the existence of a special, dedicated regulatory mechanism allowing bacteria to ensure the invariance of *free* 50S abundance.
2. The *free* 50S abundance is “high enough” so that with . Following this assumption, K’_1,50S_*_i_* happens to be almost invariant with growth rate (and saturated with respect to the *free* 50S abundance) and approximately equal to This assumption lead to a similar conclusion for K’_2,50S_*_i_*: . This alternative explanation does not necessitate the existence of a dedicated regulatory mechanism.

The in-depth analysis of the model, together with our experimental results supporting a Michaelis-Menten-like translation efficiency, led us to the conclusion that the 50S subunit abundance is either constant or not limiting for protein production. The latter conclusion is consistent with several experimental results (Antoun et al, 2006).

### A model exploring alternate orders of binding of the IFs and tRNA^fMet^ onto the 30S subunit

Milon *et al.* (2012) observed that mRNA recruitment is *in vitro* independent of the IFs and tRNA^fMet^ binding to the 30S subunit of the ribosome. Based on this observation we developed an additional model to investigate the consequences of alternate orders of binding of the IFs and tRNA^fMet^ onto the 30S subunit. The additional model includes a 5^th^ step (**step 1.2**) in between the 30S binding to the mRNA (similar to the usual **step 1** except that the R^*^*_free_* is now the only 30S subunit) and ribosome accommodation (similar to the usual **step 2** except that the R*_bi_* is now the R_IFtC_*_i_* ribosomal complex) and in which both IFs and the initiator tRNA^fMet^ bind to the R^*^*_bi_* complex (30S🞄m*_i_*). For the sake of clarity, the IFs and the initiator tRNA^fMet^ will be designated as IFtC (for IFs / tRNA^fMet^ Couple).

As compared to the general model, the specific properties of the additional model are summarized below:

| **Step 1** | ***30S binding*** | R^*^*_free_*= 30S (*free* ribosome)  m*_fi_*= *free* mRNA coded by the gene *i*  R^*^*_bi_*= 30S•m*_i_* (active ribosome)  k*_bi_*= rate of *free* ribosome binding  k_-_*_bi_*= rate of *free* ribosome release |
| --- | --- | --- |
| **Step 1.2** | ***IFtC binding to 30S🞄m_i_ complex*** | R*_IFtCi_*= 30S•IF1•IF2•IF3•tRNA^fMet^•m*_i_* complex  IFtC= IF1•IF2•IF3•tRNA^fMet^  k*_IFtCi_*= rate of IFtC binding  k_-_*_IFtCi_*= rate of IFtC release |
| **Step 2** | ***Ribosome accommodation*** | R*_ai_*=30S•IF1•IF2•IF3•tRNA^fMet^•m*_i_* (pre-initiating ribosome)  k*_ai_*= rate of ribosome positioning onto the start codon  k_-_*_a_*_i_= rate of ribosome release from the start codon |

As for the general model, we formalized the steps described above and obtained the following differential equations relating ribosome abundances and m*_i_* abundances:

|  | (**38**) |
| --- | --- |
|  | (**39**) |
|  | (**40**) |
|  | (**41**) |
|  | (**42**) |

The total mRNA for a given gene (m*_i_*) is composed of 4 species:

|  | (**43**) |
| --- | --- |

In steady-state growth the **equations** (**38**) to (**42**) lead to:

|  | (**44**) |
| --- | --- |
|  | (**45**) |
|  | (**46**) |
|  | (**47**) |
|  | (**48**) |

In order to solve this additional model, we expressed abundances of each ribosomal entity as a function of R*_ai_* abundance: , and . We can then rewrite **equation** (**43**) as a function of R*_ai_* abundance:

|  | (**49**) |
| --- | --- |

and we finally obtained after straightforward manipulations the announced result:

|  | (**50**) |
| --- | --- |

The previous expression can be easily rewritten as

|  | (**51**) |
| --- | --- |

with

As in the previous alternative model, we note that K’_2,IFtC_*_i_* can be expressed from K’_1,IFtC_*_i_*:

Interestingly, protein abundance is given by a very similar equation with aggregated K’_1,IFtC_*_i_* and K’_2,IFtC_*_i_* parameters, respectively equivalent to the K_1_*_i_* and K_2_*_i_* constants from the general model, but depending on the abundances of both initiation factors (IFs) and fMet-tRNA^fMet^. However, it is well-known that the fMet-tRNA^fMet^ abundance increases both as a relative fraction of total tRNA and in absolute abundance with increasing growth rate in *E coli* (Emilsson & Kurland, 1990) and that the abundance of each initiation factor also strongly increases with increasing growth rate in *E. coli* (Howe & Hershey, 1983). Since the translation efficiency is well represented by a Michaelis-Menten-like equation, the two K’_1,IFtC_*_i_* and K’_2,IFtC_*_i_* aggregated parameters must be near constant, which entails that abundances of neither the IFs nor the fMet-tRNA^fMet^ are limiting for ribosome assembly at any growth rates. Thus, the IFtC abundance is ‘high enough” so that with . Following this assumption, K’_1,IFtC_*_i_* happens to be almost invariant with growth rate (and saturated with respect to the IFtC abundance) and approximately equal to.

This assumption lead to a similar conclusion for K’_2,IFtC_*_i_*:

The additional model thus suggests that the variation of the 30S subunit abundance directly triggers the drop of translation efficiency from slow to fast growth.

By contrast when considering the order of binding of the IFs and tRNA^fMet^ onto the 30S subunit as described in the reduced/general model, the 4-fold decrease of R*_free_* (30S🞄IFs🞄fMet-tRNA^fMet^ initiation complex) abundance is either due to the drop of each or only one of the composing entities. Therefore, if alternative R*_free_* initiation complexes (30S🞄IFs🞄fMet-tRNA^fMet^ and 30S) coexist *in vivo*, the two nonlinear models (*i.e.* the reduced model displayed in **§2.1**, and the alternative model depicted in **§2.4**) can simultaneously be applied.

How do the Michaelis-Menten *versus* linear models of translation handle with growth-rate-dependent translation efficiency trajectories

As shown on **Appendix Figure S1**, we performed a simulation setting a lower K*_2i_* value for protein_1_ than for protein_2_ and on the condition that the *free* ribosome abundance varies with growth rate, the ratio of the translation efficiencies of these two proteins definitively varies with growth rate (note that the complete set of parameters used for the simulation is given in the legend of **Appendix Figure S1**). In this example, a growth-rate-dependent variation of R*_free_* abundance, which is common to the production of all proteins, entails that the ratio between the two theoretical proteins decreases with increasing growth rate. Such an effect cannot be obtained if the translation process is described as one irreversible step (**Appendix Figure S1C**) as in (Dressaire et al, 2009; Klumpp et al, 2009; Tadmor & Tlusty, 2008) since in this case, the translation efficiency is linearly related to the free ribosome abundance (as shown by the simulation on **Appendix Figure S1D**; red and blue dotted lines). With this simple, linear relationship, the ratio between the translation efficiencies of two proteins will therefore be invariant and independent of the free ribosome abundance (black line, **Appendix Figure S1D**). As a result, a linear model of the translation process cannot explain the variation of the ratio of the two different transcripts-related translation efficiencies observed on **Figure 3B**. Altogether our results indicated that modeling translation as an elementary three-step initiation process can reproduce the experimentally observed growth-rate-dependent translation efficiency.

# Supplementary Notes

A subset of experiments realized with the *^fbaA^*TIR strains but all experiments realized with the *^hs^*TIR strains were selected to estimate protein ratios as a function of the growth rate. Indeed, some datasets have been removed if one of the strains included in a given experiment exhibited a GFP fluorescence level close to zero. Thus, all the cultures of the *^fbaA^*TIR strains in CHG were removed because of the low GFP expression level of the *^fbaA^*TIR*_modif1_* strain. In addition, due to the variability of the fluorescence and growth acquisition, 6 additional plates were discarded from the dataset used to estimate ratios (**§3.1**). The same dataset of experiment was used to estimate the translation-related parameters (**§3.3**).

## Estimate of ratios of protein production by “minimization of mean-square error” and bootstrap

We have developed a dedicated data treatment method in order to estimate the ratios of GFP abundance between 4 strains. In view of the expected range of ratio variations, we optimized the experiment design (based on preliminary data) and developed a dedicated data treatment approach allowing to fully benefit of the experiment design. Our proposed data treatment approach had been developed in order to take advantage of the existence of replicates in the same microtiterplate and used directly the raw fluorescence datasets. Only intra-day variability has been a priori corrected due to the fact that all clones did not start growing concomitantly.

In order to estimate the ratios, we first recall that by definition, the ratios between the chosen strains satisfied this set of equations:

$$\left\{ \begin{aligned} \mathrm{RawFluo}_{1}\left( \mathrm{OD} \right)=\mathrm{AutoFluo}^{\mathrm{OD}}\left( \mathrm{OD} \right)+ \mathrm{GFP}_{1}\left( \mathrm{OD} \right) \\ \mathrm{RawFluo}_{2}\left( \mathrm{OD} \right)=\mathrm{AutoFluo}^{\mathrm{OD}}\left( \mathrm{OD} \right)+r_{2}\mathrm{GFP}_{1}\left( \mathrm{OD} \right) \\ \mathrm{RawFluo}_{3}\left( \mathrm{OD} \right)=\mathrm{AutoFluo}^{\mathrm{OD}}\left( \mathrm{OD} \right)+r_{3}\mathrm{GFP}_{1}\left( \mathrm{OD} \right) \\ \mathrm{RawFluo}_{4}\left( \mathrm{OD} \right)=\mathrm{AutoFluo}^{\mathrm{OD}}\left( \mathrm{OD} \right)+r_{4}\mathrm{GFP}_{1}\left( \mathrm{OD} \right) \\ \mathrm{RawFluo}_{5}\left( \mathrm{OD} \right)=\mathrm{AutoFluo}^{\mathrm{OD}}\left( \mathrm{OD} \right) \end{aligned} \right.$$

since the measured fluorescence (RawFluo_j_) is the sum of the GFP fluorescence (GFP_j_) and of the *B. subtilis* auto-fluorescence. In balanced growth, the GFP fluorescence is a linear function of the OD *i.e.* a_0_+a_1_ OD. $\mathrm{AutoFluo}^{\mathrm{OD}}\left( \mathrm{OD} \right)$ can be fitted by a 3^rd^-degree polynomial as already described above (Aichaoui et al, 2012; Botella et al, 2010; Buescher et al, 2012)):

AutoFluo^OD^(OD) = b_0_ +b_1_ OD+ b_2_ OD^2^ +b_3_ OD^3^

It remains to introduce a notation allowing to handle the existence of NR replicates of the j-th strain:

$$\mathrm{RawFluo}_{j}^{k}\left( \mathrm{OD} \right)=\mathrm{AutoFluo}^{\mathrm{OD}}\left( \mathrm{OD} \right)+r_{j}\mathrm{GFP}_{1}\left( \mathrm{OD} \right)$$

which corresponds to the measured fluorescence associated to the k-th replicate of strain j.

For notation convenience, we then introduced these two parameters: $r_{1}=1$ and $r_{5}=0$.

For a given a microtiterplate, the set of parameters (r_2,_ r_3_, r_4,_ a_0_, a_1_, b_0_, b_1_, b_2_ and b_3_) is estimated as the solution of the least square optimization problem (in red, parameters to be estimated):

$$\min_{\begin{aligned} \\ \begin{matrix} \\ \begin{matrix} \begin{matrix} r_{2},r_{3},r_{4} \\ a_{0},a_{1},b_{0},b_{1},b_{2},b_{3} \end{matrix} \\ r_{1}=1,r_{5}=0 \end{matrix} \end{matrix} \end{aligned}}\sum_{j=1}^{5} \sum_{k=1}^{NR(j)} \sum_{i=1}^{K(j,k)} \left\| \mathrm{RawFluo}_{j}^{k}(i)-r_{j}\left( a_{0}+a_{1}\mathrm{OD}_{j}^{k}\left( i \right) \right)-b_{0}-b_{1}\mathrm{OD}_{j}^{k}(i)-b_{2}{\mathrm{OD}_{j}^{k}}^{2}(i)-b_{3}{\mathrm{OD}_{j}^{k}}^{3}(i) \right\|^{2}$$

where NR(j) is the number of replicates associated to strain j (equal to 12 when all the replicates have satisfied all the quality control steps), K(j,k) is the number of measured points associated to the k-th replicate of strain j and finally, $\mathrm{OD}_{j}^{k}$(i) is the i-th measured OD_600_ associated to k-th replicate of strain j.

The above optimization problem does not correspond to a classical linear regression problem since it contains a product between parameters to be estimated (*i.e.* between r_j_, a_0_ and a_1_). Nevertheless, this non convex optimization problem can be solved using available and well-established approach. In view of the specific structure of the criteria, we used a classical “minimization of mean-square error” for estimating parameters (McLachlan & Krishnan, 1997).

The 95% confidence intervals for the different parameters were obtained by bootstrap (Efron, 1987).

## Computation of the translation efficiency

The measured decline of protein abundance with increasing growth rate can either be due to a drop in translation efficiency (*i.e.* rate of protein production per mRNA) or/and to growth-rate-dependent post-transcriptional processes targeting the GFP protein (Arraiano et al, 2010; Buescher et al, 2012). However, the latter hypothesis can be rejected because the GFPmut3 protein variant used in this work already proved to be (*i*) highly stable during the exponential phase of growth (Andersen et al, 1998; Botella et al, 2010) and (*ii*) a good estimator of protein abundance for a given growth rate (Buescher et al, 2012).

As described in our model (**Figure 7, Table 2**), the translation efficiency of a given gene *i* is given by:

$$\lambda_{is}=\frac{{\mu[Protein}_{is}]}{{[mRNA}_{is}]}$$

To obtain the mRNA abundance of *gfpmut3* (mRNA*_gfp j_*) from a given strain (j, among a set of strains, S), we first estimated the total mRNA abundance as function of the growth rate (**Figure 1**, **Figure EV1A,** see also **Appendix** §1.3), we then measured the distribution of the *fbaA* mRNA within the total mRNA using microarrays (see also **§1.3**) and eventually verified the ratios of *gfp* and *fbaA* mRNAs by qPCR (see also **§1.5**) as follows:

| $s\in(1,\ldots,S)$ | ${[mRNA}_{gfp s}]=\begin{matrix} \left( {[mRNA}_{tot}] \right) & \left( \frac{{[mRNA}_{fbaA}]}{{[mRNA}_{tot}]} \right) & \left( \frac{{[mRNA}_{gfp s}]}{{[mRNA}_{fbaA s}]} \right) \end{matrix}$ | | | | |
| --- | --- | --- | --- | --- | --- |
|  |  | $\begin{matrix} Nanodrop \\ Microarrays \end{matrix}$ | $Microarrays$ | $\text{qPCR}$ |  |

*fbaA* mRNA abundances in the reference strain ([mRNA*_fbaA_*]) and in the other strains ([mRNA*_fbaA s_*]) were identical (see also **§1.5**). The GFP abundance and the growth rate were calculated as previously described (see also **§1.6**). The confidence intervals were obtained by bootstrap (Efron, 1987).

## The solution space of the K_1_, K_2_ and R*_free_* parameters

The aim of this section is to detail how we estimated the *free* ribosome abundance as a function of the growth rate and of the values of the Michaelis-Menten like parameters (K_1_, K_2_) associated to the set of reporter strains.

### Mathematical framework

As described in our model (**Figure 7**), translation efficiency follows a Michaelis-Menten-like equation as a function of the R*_free_* abundance:

$\lambda_{s}(\mu_{j})=\frac{K_{1s}[R_{\mathrm{free}}(\mu_{j})]}{K_{2s}+[R_{\mathrm{free}}(\mu_{j})]}$,

where$K_{1s}{, K}_{2s}$ are the two Michaelis-Menten like parameters of the strain ‘s’, ${[R}_{free}(\mu_{j})]$ corresponds to the R*_free_* abundance at growth rate, $\mu_{j}$ with $j\in\left( 1,\ldots,n \right),$ and n stands for the number of the different growth rate measurements. We will show in the sequel that the problem can be solved if (and only if) we can measure the translation efficiency of at least two reporter strains with different Michaelis-Menten like parameters in two different growth rate conditions. The strains used to solve the problem will thus be chosen in order to satisfy these conditions.

In this section, the estimation problem is introduced from a theoretical viewpoint while practical purposes are presented in the next section. Let us introduce two Michaelis-Menten-like functions standing for the translation efficiency of two strains *l* and *m*:

| $j\in(1,\ldots,n)$ | $\lambda_{l}(\mu_{j})=\frac{K_{1l}[R_{free}(\mu_{j})]}{K_{2l}+[R_{free}(\mu_{j})]}$ and $\lambda_{m}\left( \mu_{j} \right)=\frac{K_{1m}\left[ R_{free}\left( \mu_{j} \right) \right]}{K_{2m}+\left[ R_{free}\left( \mu_{j} \right) \right]}$ |
| --- | --- |

In the above equations${[R}_{free}(\mu_{j})$] stands for the R*_free_* abundance as a function of the growth rate $\mu_{j}$. Without loss of generality, we rewrote the two functions in a more convenient way

| $j\in(1,\ldots,n)$ | $\lambda_{l}(j)=\frac{Q_{1l}}{Q_{2l}+z(j)}$ and $\lambda_{m}(j)=\frac{Q_{1m}}{Q_{2m}+z(j)}$ |
| --- | --- |

with $z(j)=\frac{1}{[R_{free}(\mu_{j})]}$, $Q_{1l}=\frac{K_{1l}}{K_{2l}}$, $Q_{2l}=\frac{1}{K_{2l}}$,$Q_{1m}=\frac{K_{1m}}{K_{2m}}$ and $Q_{2m}=\frac{1}{K_{2m}}$.

The general estimation problem can be set as the following:

| $\min_{\begin{aligned} \\ \begin{matrix} \\ \begin{matrix} \begin{matrix} Q_{1l}, Q_{2l}, Q_{1m}, Q_{2m} \\ z\left( 1 \right), z\left( 2 \right), \ldots, z(n) \end{matrix} \\ \end{matrix} \end{matrix} \end{aligned}} \sum_{j=1}^{n} \left( \left\Vert\lambda_{l}\left( j \right)-\frac{Q_{1l}}{Q_{2l}+z(j)} \right\Vert^{2}+\left\Vert\lambda_{m}\left( j \right)-\frac{Q_{1m}}{Q_{2m}+z(j)} \right\Vert^{2} \right)$ | (**52**) |
| --- | --- |

where translation efficiencies associated to strain *l* and *m* *i.e.* {($\lambda_{l}(j)$, $\lambda_{m}(j)$} is assumed to be measurable for each growth rate condition {$\mu_{j}$} with $j\in\left( 1,\ldots,n \right).$

### The general estimation problem has an infinite number of equivalent solutions

Unfortunately, this problem (**Equation (52)**) has an infinite number of equivalent solutions. Indeed, if (Q_1_*_l_*, Q_2_*_l_*, Q_1_*_m_*, Q_2_*_m_*) and {z(j)} is a solution of the general estimation problem (**52)** then the set of parameters defined by

| $\left\{ \begin{aligned} \tilde{Q}_{1l}= \beta\frac{Q_{1l}}{\alpha} \\ \tilde{Q}_{2l}={\beta Q}_{2l} \\ \tilde{Q}_{1m}=\beta\frac{Q_{1m}}{\alpha} \\ \tilde{Q}_{2m}=\beta\left( \frac{Q_{2m}-Q_{2l}}{\alpha}+Q_{2l} \right) \\ \tilde{z}\left( j \right)=\beta\left( \frac{z\left( j \right)}{\alpha}{+Q}_{2l}\frac{1-\alpha}{\alpha} \right) for j \in(1,..,n) \end{aligned} \right.$ | (**53**) |
| --- | --- |

is also a set of solution of the general estimation problem (**52)** for any non-zero value of scaling parameters α and β.

The case α=1 has some interesting features. Indeed, in this case, the set of equivalent solutions is given by (βQ_1_*_l_*, βQ_2_*_l_*, βQ_1_*_m_*, βQ_2_*_m_*) and {βz(j)} for any $\beta\neq0$: the estimation problem is then “insensitive to the units of R*_free_* abundance”. More practically, it means that an absolute quantification of the R*_free_* abundance cannot be obtained without supplementary information. Additional constraints of positivity on all parameters can be included in the estimation problem (**52)**. These new constraints only restrict the set of possible scaling values, since in this case β > 0 and $\alpha$ has to belong to the following interval$[\min\left( 0,1-\frac{Q_{2m}}{Q_{2l}} \right),1]$. Nevertheless, this restriction does not remove the infinite number of equivalent solutions to the estimation problem.

### An equivalent estimation problem with a unique solution (when exists)

Here we introduce an “equivalent estimation problem” corresponding to problem (**52)** where two free parameters have been set. Any solution of the general estimation problem can be deduced from the solution of the equivalent estimation problem. As for example, if $\tilde{Q}_{1m}(>0)$ and $\tilde{Q}_{2l}(>0)$ are set and if we assume that the equivalent problem has a solution given by ($\tilde{Q}_{1l}\tilde{Q}_{2l}\tilde{Q}_{1m}\tilde{Q}_{2m})$and {$\tilde{z}\left( j \right)$}, then any solution of the estimation problem (**52)** is related to the solution of the equivalent problem by the following equations:

$$\left\{ \begin{aligned} Q_{1l}= \gamma\tilde{Q}_{1l} \\ Q_{2l}=\theta\tilde{Q}_{2l} \\ Q_{1m}=\gamma\tilde{Q}_{1m} \\ Q_{2m}=\gamma\tilde{Q}_{2m}+(\theta-\gamma)\tilde{Q}_{2l} \\ z\left( j \right)=\gamma\tilde{z}\left( j \right)+ (\theta-\gamma)\tilde{Q}_{2l} \end{aligned} \right.$$

with $\gamma$ and $\theta$ are two non-zero constants

### The equivalent estimation problem has always a (unique) solution

Let us now briefly discuss the question of (a) the existence of a solution for the equivalent estimation problem and of (b) the identifiability of parameters. For identifiability, we assumed that measurements are not corrupted by any noise.

The knowledge of the translation efficiency of two strains in two different growth rates conditions is enough for identifying (theoretically) the model parameters. In this case, we have six parameters in **Equation** (**52)**, *i.e.* Q_1_*_l_*, Q_2_*_l_*, Q_1_*_m_*, Q_2_*_m_*, z(1) and z(2), and two of them are set to *a priori* values in the equivalent estimation problem. Thus, four model parameters remain to be estimated. The estimation problem is equivalent to the resolution of a linear system of equations with four equality constraints depending on four unknown parameters which has always a (unique) solution, *i.e.,* the system parameters are identifiable.

**Remarks**

- If we add a new growth-rate condition, then a supplementary parameter, *i.e.* ‘z(j+1)’ where ‘j+1’ is the new growth-rate condition, has to be estimated. Addition of z(j+1) leads to include two supplementary equality constraints (one for each strain) on the equivalent estimation problem. From a theoretical viewpoint, we have more constraints than parameters and thus a net gain to use a large set of growth-rate conditions.
- The identifiability result remains true when more than two strains are considered. Like in the case of two strains, only two parameters have to be set in order to have a unique solution (allowing the parameterization of all the solutions of the general estimation problem).

### Resolution of the equivalent estimation problem with noisy data

When data are noisy, the equivalent estimation problem is not equivalent to a linear problem and we have then to solve the following optimization problem:

| $\min_{\begin{aligned} \\ \begin{matrix} \\ \begin{matrix} \begin{matrix} Q_{1l}, Q_{2l}, Q_{1m}, Q_{2m} \\ z\left( 1 \right), z\left( 2 \right), \ldots, z(n) \end{matrix} \\ \end{matrix} \end{matrix} \end{aligned}} \sum_{j=1}^{n} \left( \left\Vert\lambda_{l}\left( j \right)-\frac{Q_{1l}}{Q_{2l}+z(j)} \right\Vert^{2}+\left\Vert\lambda_{m}\left( j \right)-\frac{Q_{1m}}{Q_{2m}+z(j)} \right\Vert^{2} \right)$ | (**54**) |
| --- | --- |

where the translation efficiencies of to strain *l* and *m* *i.e.* {($\lambda_{l}(j)$, $\lambda_{m}(j)$} are assumed to be available for each growth rate conditions {$\mu_{j}$} with $j\in\left( 1,\ldots,n \right)$and where two parameters are supposed to be fixed.

We therefore solved the optimization problem through a classical Gauss-Newton like algorithm. Unfortunately, since the considered cost function is not convex, local minima exist. In order to obtain the global minimum, we then solved the minimization problem (**Equation (53)**) for different initial conditions, *i.e.* in the case of two growth rate conditions, four initial values associated to the four parameters to be identified. We finally kept the solution corresponding to the minimal value of the cost function.

### Handling a large set of "growth rate" conditions

A large set of growth-rate conditions theoretically leads to add new constraints on the parameter estimation (see section d). However, for each new growth condition integrated, a additional parameter needs to be estimated. Thus, increasing the set of growth rate conditions leads to some computational difficulties due to the curse of parameter dimension (since we have to sample enough initial conditions in order to reach the global minimum).

In order to use a large set of conditions (n>4), we then assumed that the *free* ribosome abundance (variable z), is a polynomial function of the growth rate:

| $z= \gamma_{0}+ \gamma_{1}\mu\mu+\gamma_{2} \mu^{2}+\gamma_{3} \mu^{3}$ | (**55**) |
| --- | --- |

We have now to solve this new minimization problem:

| $\min_{\begin{aligned} \\ \begin{matrix} \\ \begin{matrix} \begin{matrix} Q_{1l}, Q_{2l}, Q_{1m}, Q_{2m} \\ {\gamma_{0,}\gamma}_{1} , \gamma_{2},\gamma_{3} \end{matrix} \\ \end{matrix} \end{matrix} \end{aligned}} \sum_{j=1}^{n} \left( \left\Vert\lambda_{l}\left( j \right)-\frac{Q_{1l}}{Q_{2l}+\sum_{k=0}^{3} \gamma_{k} \mu_{j}^{k}} \right\Vert^{2}+\left\Vert\lambda_{m}\left( j \right)-\frac{Q_{1m}}{Q_{2m}+\sum_{k=0}^{3} \gamma_{k} \mu_{j}^{k}} \right\Vert^{2} \right)$ | (**56**) |
| --- | --- |

where two parameters among $Q_{1l}, Q_{2l}, Q_{1m}\mathrm{and}Q_{2m}$ have been fixed.

### Design and use of dedicated reporter strains to overcome the infinite number of equivalent solutions

From a biological viewpoint, we want to estimate the amplitude of the *free* ribosome variation and its specific shape with respect to the growth rate. To do so, we have then to fix two parameters among $Q_{1l}, Q_{2l}, Q_{1m}\mathrm{and}Q_{2m}$. $Q_{1l}$ or $Q_{1m}$ can be fixed to 1. None of these parameters has an effect on the shape or on the amplitude of the *free* ribosome variation with respect to the growth rate. By contrast, fixing the second parameter ($Q_{2l}$ or $Q_{2m}$) has necessarily an impact on the estimation of R*_free_* and parameters since implicitly $\alpha$ and $\beta$ are now fixed in Equation (57). Nevertheless, $Q_{2l}$ or $Q_{2m}$ needs to be fixed in order to obtain a “conservative estimation of the maximum variation of R*_free_*”. That means that the real variation of R*_free_* as a function of the growth rate has to be greater than or equal to the one obtained with the fixed parameters. We first selected the synthetic strain ‘j’ among all the synthetic strains having the largest translation efficiency ratio between the lowest and the largest growth rate. We then set for this strain Q_1j_= 1 and Q_2j_= 0.

### Implication of a possible error induced by an inaccurate Q_2j_ value

We now discuss the consequences of a possible discrepancy on the fixed parameter “Q*_2j_*” on the estimation of the *free* ribosome variation. For the sake of simplicity, we only considered 2 strains (the general case can be handled in the same way). We assume that we have obtained using our *a priori* choice of parameters ($Q_{1m}$=1 and Q_2m_= 0) and after the estimation step the following solution:

| $\lambda_{l}=\frac{Q_{1l}}{Q_{2l}+z}$ and $\lambda_{m}=\frac{1}{z}$ |  |
| --- | --- |

Now, we assume that Q_2m_ is not equal to zero but to a given value $\tilde{Q}_{2m}>0$.

In this case, we obtained the following “true” solution:

| $\lambda_{l}=\frac{\tilde{Q}_{1l}}{\tilde{Q}_{2l}+\tilde{z}}$ and $\lambda_{m}=\frac{1}{\tilde{Q}_{2m}+\tilde{z}}$ |  |
| --- | --- |

But, we already pointed out that the parameters of the solution **(52)** and the ones of the true solution are related by relation **Equation** (**53)**. Thus, we have:

$$\left\{ \begin{aligned} \tilde{Q}_{1l}=Q_{1l} \\ \tilde{Q}_{2l}={\beta Q}_{2l} \\ 1=\frac{\beta}{\alpha} \\ \tilde{Q}_{2m}=Q_{2l}(\beta-1) \\ \tilde{z}=z-\tilde{Q}_{2m} \end{aligned} \right.$$

The “true curve” is only shifted down by a constant term and the shape of $\tilde{z} (\mu)$ and $z(\mu)$ are kept.

Moreover, the maximum amplitude of variation of the estimated curve is

$\mathrm{Max}_{\mathrm{Ratio}}=\frac{\max_{(\mu\in[0.25;1.4]))} \left( \frac{1}{z\left( \mu\right)} \right)}{\min_{(\mu\in[0.25;1.4]))} \left( \frac{1}{z\left( \mu\right)} \right)}=\frac{\frac{1}{z_{min}}}{\frac{1}{z_{max}}}=\frac{z_{max}}{z_{min}}$,

where $z_{max}=\max_{(\mu\in[0.25;1.4]))} \left( z\left( \mu\right) \right)$ and $z_{min}=\min_{(\mu\in[0.25;1.4]))} \left( z\left( \mu\right) \right)$.

For the true solution, we have then

$\tilde{Max}_{\mathrm{ratio}}=\frac{\max_{(\mu\in[0.25;1.4]))} \left( \frac{1}{\tilde{z}\left( \mu\right)} \right)}{\min_{(\mu\in[0.25;1.4]))} \left( \frac{1}{\tilde{z}\left( \mu\right)} \right)}=\frac{\frac{1}{\tilde{z}_{min}}}{\frac{1}{\tilde{z}_{max}}}=\frac{z_{max}-\tilde{Q}_{2m}}{z_{min}-\tilde{Q}_{2m}}$,

Therefore, we can deduce that $\mathrm{Max}_{\mathrm{Ratio}} \leq\tilde{Max}_{\mathrm{ratio}}$ since we have

$$\frac{z_{max}-\tilde{Q}_{2m}}{z_{min}-\tilde{Q}_{2m}}-\frac{z_{max}}{z_{min}}=\frac{\tilde{Q}_{2m}(z_{max}-z_{min})}{{(z}_{min}-\tilde{Q}_{2m})z_{min}}\geq0$$

and the true value of *free* ribosome abundance $\tilde{z}_{min}$ (=$z_{min}-\tilde{Q}_{2m})$ is positive by definition.

To summarize, using a prefixed value of Q_2_ for specific strains leads to a conservative estimation of the maximum variation of R*_free_* abundance and of the shape of the curve with respect to the growth rate.

### Statistical treatment

Regarding the specificities of our data treatment, we used the bootstrap approach (Efron, 1987) for characterizing all the statistical properties of our estimation scheme.

## Genome-wide estimation of R*_free_* abundance and of the {K_1_*_i_*, K_2_*_i_*} pairs for each transcript

### Genome-wide estimation of {K_1_*_i_*, K_2_*_i_*} pairs for each transcript

We developed a dedicated two-step iterative procedure for the estimation of {K_1_*_i_*, K_2_*_i_*} for a large range of transcripts of *B. subtilis* combining different datasets in five conditions: RNA quantification and DNA microarrays (obtained in this study), absolute protein quantification (Muntel et al. 2014, Goelzer et al 2015). We first computed for each condition (index *j*) and each transcript (index *i*) the translation efficiency as:

$$\lambda_{ji}=\frac{\mu_{j}P_{ji}}{m_{ji}}$$

where $\mu_{j}$, $P_{ji}$, $m_{ji}$, denotes in condition *j* the measured growth rate, the measured absolute abundances of protein *i* and mRNA *i* respectively. To infer {K_1_*_i_*, K_2_*_i_*}, we first selected the transcripts for which we had determined the translation efficiency in at least 3 conditions.

During the first step of the procedure, we identified the parameters {K_1_*_i_*, K_2_*_i_*} for each transcript by solving the following optimization problem:

| $\min_{\begin{aligned} \\ \begin{matrix} \\ \begin{matrix} \begin{matrix} Q_{1i}, Q_{2i}, \\ \end{matrix} \\ \end{matrix} \end{matrix} \end{aligned}} \sum_{j=1}^{n} \left( \left\Vert\lambda_{i}\left( j \right)-\frac{Q_{1i}}{Q_{2i}+\sum_{k=0}^{3} \gamma_{k} \mu_{j}^{k}} \right\Vert^{2} \right)$ |  |
| --- | --- |
|  |  |

where the coefficients ${\gamma_{0,}\gamma}_{1} , \gamma_{2},\gamma_{3}$ are fixed to the values identified during step 2. Here *n* represents the number of conditions for which we have determined the translation efficiency (between 3 and 5 conditions).

During the second step of the procedure, we identified the parameter R*_free_* by solving the following optimization problem for fixed values ${(Q}_{1i},Q_{2i})$ that were determined during step 1:

$$\min_{\begin{aligned} \\ \begin{matrix} \\ \begin{matrix} \begin{matrix} {\gamma_{0,}\gamma}_{1} , \gamma_{2},\gamma_{3}, \\ \end{matrix} \\ \end{matrix} \end{matrix} \end{aligned}} \sum_{i=1}^{p} \sum_{j=1}^{n} \left( \left\| \lambda_{i}\left( j \right)-\frac{Q_{1i}}{Q_{2i}+\sum_{k=0}^{3} \gamma_{k} \mu_{j}^{k}} \right\|^{2} \right)$$

Here the number p corresponds to the number of transcripts that are selected for the estimation of R*_free_*. We repeated the whole procedure several times until the R*_free_* parameter converged, i.e. $\sum_{k=0}^{3} \left\| \gamma_{k}^{t}-\gamma_{k}^{t-1} \right\|\leq\varepsilon$.

Unfortunately, since the considered cost functions of the two optimization problems are not convex, local minima exist. In order to obtain the global minimum, we then solved at each iteration, each minimization problem for different initial conditions of the parameters. We finally kept the solution corresponding to the minimal value of the cost function.

### R*_free_* abundance estimation for the synthetic strains *versus* the genome scale proteome

For the genome-scale proteome, we applied the same procedure than the one for synthetic strains. We fixed two parameters in order to estimate R*_free_* abundance and the parameters of each protein. Following the derivations presented in the previous sections, we selected the protein having the largest translation efficiency ratio between the lowest and the largest growth rate. We set for this specific protein ‘m’ Q_1m full_ = 1 and Q_2m full_= 0. This ratio is larger than the one of the synthetic strain ‘j’ (in 3.3.1.7). Therefore, following the demonstration of section 3.3.1.8, the variation of R*_free_* abundance that is estimated using the genome-scale proteome will necessarily be larger than the one obtained with the set of synthetic strains. We can furthermore deduced that each parameter Q_2*_ estimated on the set of synthetic strains is slightly underestimated compared to the real ones.

## Higher R*_free_* abundance at slow than at fast growth should ensure that slowly growing cells are prone to adaptation

Fine optimization of resource allocation is usually not compatible with fast adaptation unless cells are prone to metabolically respond to the nutritional transition (Buescher et al, 2012). However, during nutritional upshifts, both the intracellular macromolecular composition and the growth rate exhibit fast variations (Kjeldgaard et al, 1958). In bacteria, two coupled key metabolites, GTP and (p)ppGpp, coordinate the global adaptive response to newly available environmental resources. After an upshift of nutriments, there is an increase of resources inside the cell and through a RelA dedicated mechanism: the amount of (p)ppGpp decreases, and the inhibitory effect of ppGpp on the production of GTP decreases and leads to an increase of the GTP abundance. On the one hand, the transcription of rRNA and of other GTP-dependent promoters is stimulated by the growth rate-dependent increases in GTP abundance and in RNA polymerase activity (Klumpp & Hwa, 2008; Krasny & Gourse, 2004). On the other hand, an increase of resources inside the cell combined with an elevated GTP abundance enhances translation initiation by stimulating the hydrolysis of the IF2-bound GTP that allows the conformational change of the ribosomal 30S subunit (Milon et al; Milon et al). By contrast, ppGpp binding to IF2 leads to translation inhibition. Thus, the abundance of R*_free_* (30S🞄mRNA🞄IFs🞄**GTP**🞄fMet-tRNA^fMet^) can rapidly vary along with variations in the levels of GTP and ppGpp (assuming that the fMet-tRNA^fMet^ is not limiting). During a nutritional upshift, both the R*_free_* abundance and increase in GTP abundance will facilitate fast adaptation. In particular, the elevated abundance of *free* ribosome during slow growth, which could be regarded as a loss of energy, supports rapid production of the proteins required for cellular adaptation. By contrast, at fast growth, an overabundance of *free* ribosome is unnecessary. The nonlinearity of protein production offers a broad repertoire of growth rate-dependent protein expression levels for optimal transient adaptation. The next question to address is whether evolutionary constraints operated at the level of the selective growth rate-dependent regulation of bacterial translation to optimize dynamic cell adaptation, and not only to optimize steady-state growth.

# Supplementary Tables

Table S1: Protein productions in poor and rich growth conditions.

| Relative protein production^*^ | | |
| --- | --- | --- |
| Strain | S medium^†^ (µ=0.6 h^-1^) | CHG medium^†^ (µ=1.6 h^-1^) |
| OB02 | 2.8 | 2.0 |
| OB11 | 1.5 | 1.8 |
| OB04 | **1.0** | **1.0** |
| OB06 | 0.9 | 1.0 |
| OB10 | 0.7 | 0.6 |
| OB03 | 0.5 | 0.3 |
| OB08 | 0.2 | 0.1 |
| OB05 | 0.1 | *<0.1* |
| OB07 | *<0.1* | *<0.1* |
| OB09 | *<0.1* | *<0.1* |
| OB12 | 3.0 | 2.4 |
| OB21 | 1.3 | 1.6 |
| OB14 | **1.0** | **1.0** |
| OB16 | 1.0 | 1.0 |
| OB20 | 1.0 | 0.9 |
| OB13 | 0.7 | 0.6 |
| OB18 | 0.3 | 0.2 |
| OB15 | 0.2 | 0.1 |
| OB17 | *<0.1* | *<0.1* |
| OB19 | *<0.1* | *<0.1* |

^*^ Translational efficiency corresponds to the ratio of the normalized GFP abundance to the abundance of the *^fbaA^*TIR*_fbaA_* and *^hs^*TIR*_fbaA_* strains. Error is ± 0.1.

^†^ Media are described in the **SI** §1.

Table S2: Plasmids and Primers used in this work.

| **Plasmids** | | **Primers for pBSBII amplification*** | **Primers for promoter amplification*** |
| --- | --- | --- | --- |
| pFB0 | **F_0_:** GGGAAGGAGGTGATCCAATGCGTA AAGGAGAAGAACTTTT | | **F*_UNIV1_*:** CCGCGGGCTTTCCCAGCCG GCATTCGTATTTTGCTAA |
|  | **R*_UNIV1_*:** GGGAAAGCCCGCGGTAAAAG | | **R*_fbaA_*_01_:** CCTCCTTCCCACCTGTAGC CTGATTGTCTTCTAGCG |
| pFB1 | **F*_modif1_*:** GGGAAGGAGGTGATCCAGTAT GCGTAAAGGAGAAGAACTTTT | | **F*_UNIV1_*** |
|  | **R*_UNIV1_*** | | **R*_fbaA_*_01_** |
| pFB2 | **F*_fbaA_*:** GGGAAGGAGGACATTCGACATG CGTAAAGGAGAAGAACTTTT | | **F*_UNIV1_*** |
|  | **R*_UNIV1_*** | | **R*_fbaA_*_24_:** TGTCCTCCTTCCCACCTGT AGCCTGATTGTCTTCTAGCG |
| pFB3 | **F*_modif2_*:** GGGAAGGGGGACATTCGACAT GCGTAAAGGAGAAGAACTTTT | | **F*_UNIV1_*** |
|  | **R*_UNIV1_*** | | **R*_fbaA_*_356_:** TGTCCCCCTTCCCACCT GTAGCCTGATTGTCTTCTAGCG |
| pFB4 | **F_4_:** GGGAAGGAGGACATTCGACGTGC GTAAAGGAGAAGAACTTTT | | **F*_UNIV1_/*R*_fbaA_*_24_** |
|  | **R*_UNIV1_*** | |  |
| pFB5 | **F_5_:** GGGAAGGGGGACATTCGACTTG CGTAAAGGAGAAGAACTTTT | | **F*_UNIV1_/*R*_fbaA_*_356_** |
|  | **R*_UNIV1_*** | |  |
| pFB6 | **F_6_:** GGGAAGGGGGACATTCGACGTGC GTAAAGGAGAAGAACTTTT | | **F*_UNIV1_/*R*_fbaA_*_356_** |
|  | **R*_UNIV1_*** | |  |
| pFB7 | **F_7_:** GGGAAGGGCGACATTCGACGTGCG TAAAGGAGAAGAACTTTT | | **F*_UNIV1_*** |
|  | **R*_UNIV1_*** | | **R*_fbaA_*_7:_** TGTCGCCCTTCCCACCTGTA GCCTGATTGTCTTCTAGCG |
| pFB8 | **F_8_:** GGGAAGGAGGGGGTTCGACATGCG TAAAGGAGAAGAACTTTT | | **F*_UNIV1_*** |
|  | **R*_UNIV1_*** | | **R*_fbaA_*_8_:** GGGAAGGAGGGGGTTCGACA TGCGTAAAGGAGAAGAACTTTT |
| pHS0 | **F_0_*/*R*_UNIV1_*** | | **F*_hs_*_01_*:*** CCTCCTTCCCACCAATTGTTA TCCGCTCACAATTAC |
|  |  | | **R*_UNIV2_:*** CGCGGGCTTTCCCAGCCAT GAGAATTCGACTCTCTA |
| pHS1 | **F*_modif1_/*R*_UNIV1_*** | | **F*_hs_*_01_*/*R*_UNIV2_*** |
| pHS2 | **F*_fbaA_/* R*_UNIV1_*** | | **F*_hs_*_24_:** TGTCCCCCTTCCCACCAATT GTTATCCGCTCACAATTAC |
|  |  | | **R*_UNIV2_*** |
| pHS3 | **F*_modif2_/* R*_UNIV1_*** | | **F*_hs_*_356_:** TGTCGCCCTTCCCACCAATT GTTATCCGCTCACAATTAC |
|  |  | | **R*_UNIV2_*** |
| pHS4 | **F_4_*/*R*_UNIV1_*** | | **F*_hs_*_24_*/* R*_UNIV2_*** |
| pHS5 | **F_5_*/*R*_UNIV1_*** | | **F*_hs_*_356_*/* R*_UNIV2_*** |
| pHS6 | **F_6_*/*R*_UNIV1_*** | | **F*_hs_*_356_/R*_UNIV2_*** |
| pHS7 | **F_7_*/* R*_UNIV1_*** | | **F*_hs_*_7_:** CCCCCTCCTTCCCACCAATTG TTATCCGCTCACAATTAC |
|  |  | | **R*_UNIV2_*** |
| pHS8 | **F_8_*/* R*_UNIV1_*** | | **F*_hs_*_8_:** GTTCCTCCTTCCCACCAATTG TTATCCGCTCACAATTAC |
|  |  | | **R*_UNIV2_*** |
| pHS9 |  | | **F*_hs9_*:** CCGCGGGCTTTCCCAGCCATG AGAATTCGACTCTCTA |
|  |  | | **R*_UNIV2_*** |

* F stands for forward and R for reverse primers.

Table S3: Strains used in this work.

| **Strain** | **Relevant Genotype** | **Plasmid**→**Strain*** |
| --- | --- | --- |
|  |  |  |
| OB01 | *ΔamyE*::(*lacI* P*_hs_*/*cm*) | pOB1→BSB168 |
| OB02 | *ΔamyE*::(*lacI* P*_hs_*/*cm*), P*_fbaA_*TIR_0_*gfpmut3*/spec | pFB0→BSOB01 |
|  |  |  |
| OB03 | *ΔamyE*::(*lacI* P*_hs_*/*cm*), P*_fbaA_*TIR*_modif1_gfpmut3*/spec | pFB1→BSOB01 |
|  |  |  |
| OB04 | *ΔamyE*::(*lacI* P*_hs_*/*cm*), P*_fbaA_*TIR*_fbaA_gfpmut3*/spec | pFB2→BSOB01 |
|  |  |  |
| OB05 | *ΔamyE*::(*lacI* P*_hs_*/*cm*), P*_fbaA_*TIR *_modif2_gfpmut3*/spec | pFB3→BSOB01 |
|  |  |  |
| OB06 | *ΔamyE*::(*lacI* P*_hs_*/*cm*), P*_fbaA_*TIR_4_*gfpmut3*/spec | pFB4→BSOB01 |
|  |  |  |
| OB07 | *ΔamyE*::(*lacI* P*_hs_*/*cm*), P*_fbaA_*TIR_5_*gfpmut3*/spec | pFB5→BSOB01 |
|  |  |  |
| OB08 | *ΔamyE*::(*lacI* P*_hs_*/*cm*), P*_fbaA_*TIR_6_*gfpmut3*/spec | pFB6→BSOB01 |
|  |  |  |
| OB09 | *ΔamyE*::(*lacI* P*_hs_*/*cm*), P*_fbaA_*TIR_7_*gfpmut3*/spec | pFB7→BSOB01 |
|  |  |  |
| OB10 | *ΔamyE*::(*lacI* P*_hs_*/*cm*), P*_fbaA_*TIR_8_*gfpmut3*/spec | pFB8→BSOB01 |
|  |  |  |
| OB11 | *ΔamyE*::(*lacI* P*_hs_*/*cm*), P*_fbaA_*TIR*_short_gfpmut3*/spec | BBA0016^†^ →BSOB01 |
| OB12 | *ΔamyE*::(*lacI* P*_hs_*TIR_0_*gfpmut3*/spec P*_hs_*/*cm*) | pHS0→BSOB01 |
|  |  |  |
| OB13 | *ΔamyE*::(*lacI* P*_hs_*TIR*_modif1_gfpmut3*/spec P*_hs_*/*cm*) | pHS1→BSOB01 |
|  |  |  |
| OB14 | *ΔamyE*::(*lacI* P*_hs_*TIR*_fbaA_gfpmut3*/spec P*_hs_*/*cm*) | pHS2→BSOB01 |
|  |  |  |
| OB15 | *ΔamyE*::(*lacI* P*_hs_*TIR *_modif2_gfpmut3*/spec P*_hs_*/*cm*) | pHS3→BSOB01 |
|  |  |  |
| OB16 | *ΔamyE*::(*lacI* P*_hs_*TIR_4_*gfpmut3*/spec P*_hs_*/*cm*) | pHS4→BSOB01 |
|  |  |  |
| OB17 | *ΔamyE*::(*lacI* P*_hs_*TIR_5_*gfpmut3*/spec P*_hs_*/*cm*) | pHS5→BSOB01 |
| OB18 | *ΔamyE*::(*lacI* P*_hs_*TIR_6_*gfpmut3*/spec P*_hs_*/*cm*) | pHS6→BSOB01 |
| OB19 | *ΔamyE*::(*lacI* P*_hs_*TIR_7_*gfpmut3*/spec P*_hs_*/*cm*) | pHS7→BSOB01 |
|  |  |  |
| OB20 | *ΔamyE*::(*lacI* P*_hs_*TIR_8_*gfpmut3*/spec P*_hs_*/*cm*) | pHS8→BSOB01 |
|  |  |  |
| OB21 | *ΔamyE*::(*lacI* P*_hs_*TIR*_short_gfpmut3*/spec P*_hs_*/*cm*) | pHS9→BSOB01 |
|  |  |  |

^*^ Plasmids used for transformation (→) of either BSB168 or BSOB1 strains.

^†^ Genomic DNA from Buescher, J.M.*, et al.* Global network reorganization during dynamic adaptations of *Bacillus subtilis* metabolism. *Science* 335, 1099-1103 (2012). used for transformation (→).

Supplementary Figures

Figure S1. The translation process is well described by a Michaelis-Menten like equation.

(A) Knowledge-based, three-step translation initiation model in four reversible and irreversible steps (for more information, see Appendix §2.1). The growth rate-dependent parameters of the model are the abundance of the translation initiation complex (R*_free_*), the mRNA abundance (mRNA*_i_*), and the rate of elongation (k*_pi_*, (Bremer & Dennis, 2008)). The growth rate-independent parameters of the model are the binding constant of R*_free_* onto the mRNA*_i_* (k*_bi_*), the release constant (k*_-bi_*) of mRNA*_i_*-associated R*_free_* (R*_bi_*), the accommodation constant (k*_ai_*) of R*_bi_* on the start codon, the disaccommodation constant (k*_-ai_*) of R*_bi_*-accommodated R*_ai_* from the start codon, and the constant of the initiation of translation elongation (k*_ti_*). (B) Prediction of the translation efficiencies with increasing *free* ribosome abundance for two theoretical proteins with a constant K_11_=120, K_21_=2 (red dotted line) and K_12_=100, K_22_=10 (blue dotted line). The ratio between the translation efficiencies (black line) is dependent on the *free* ribosome abundance. (C) Current model of the translation process in two irreversible steps. The growth rate-dependent parameters of the model are the abundance of the translation initiation complex (R*_free_*), the mRNA abundance (mRNA*_i_*), and the rate of elongation (k*_pi_*). The growth rate-independent parameters of the model is the binding constant of R*_free_* onto the mRNA*_i_* (k*_i_*) (D) Prediction of the translation efficiencies with increasing *free* ribosome abundance for two theoretical proteins with a constant k_1_=10 (red dotted line) and k_2_=5 (blue dotted line). The ratio between the translation efficiencies (black line) is independent of the *free* ribosome abundance.

# Supplementary references

Andersen JB, Sternberg C, Poulsen LK, Bjorn SP, Givskov M, Molin S (1998) New unstable variants of green fluorescent protein for studies of transient gene expression in bacteria. *Appl Environ Microbiol* **64:** 2240-2246

Antoun A, Pavlov MY, Lovmar M, Ehrenberg M (2006) How initiation factors tune the rate of initiation of protein synthesis in bacteria. *EMBO J* **25:** 2539-2550

Arraiano CM, Andrade JM, Domingues S, Guinote IB, Malecki M, Matos RG, Moreira RN, Pobre V, Reis FP, Saramago M, Silva IJ, Viegas SC (2010) The critical role of RNA processing and degradation in the control of gene expression. *FEMS Microbiol Rev* **34:** 883-923

Efron B (1987) Better Bootstrap Confidence Intervals. *Journal of the American Statistical Association* **82:** 171-185

Emilsson V, Kurland CG (1990) Growth rate dependence of transfer RNA abundance in *Escherichia coli*. *EMBO J* **9:** 4359-4366

Goelzer A, Bekkal Brikci F, Martin-Verstraete I, Noirot P, Bessieres P, Aymerich S, Fromion V (2008) Reconstruction and analysis of the genetic and metabolic regulatory networks of the central metabolism of *Bacillus subtilis*. *BMC Syst Biol* **2:** 20

Gur E, Biran D, Ron EZ (2011) Regulated proteolysis in Gram-negative bacteria--how and when? *Nat Rev Microbiol* **9:** 839-848

Howe JG, Hershey JW (1983) Initiation factor and ribosome levels are coordinately controlled in *Escherichia coli* growing at different rates. *J Biol Chem* **258:** 1954-1959

Jayapal KP, Sui S, Philp RJ, Kok YJ, Yap MG, Griffin TJ, Hu WS (2010) Multitagging proteomic strategy to estimate protein turnover rates in dynamic systems. *J Proteome Res* **9:** 2087-2097

Jules M, Le Chat L, Aymerich S, Le Coq D (2009) The *Bacillus subtilis ywjI* (*glpX*) gene encodes a class II fructose-1,6-bisphosphatase, functionally equivalent to the class III Fbp enzyme. *J Bacteriol* **191:** 3168-3171

Kjeldgaard NO, Maaloe O, Schaechter M (1958) The transition between different physiological states during balanced growth of *Salmonella typhimurium*. *J Gen Microbiol* **19:** 607-616

Kock H, Gerth U, Hecker M (2004) The ClpP peptidase is the major determinant of bulk protein turnover in *Bacillus subtilis*. *J Bacteriol* **186:** 5856-5864

Krasny L, Gourse RL (2004) An alternative strategy for bacterial ribosome synthesis: *Bacillus subtilis* rRNA transcription regulation. *EMBO J* **23:** 4473-4483

Kunst F, Ogasawara N, Moszer I, Albertini AM, Alloni G, Azevedo V, Bertero MG, Bessieres P, Bolotin A, Borchert S, Borriss R, Boursier L, Brans A, Braun M, Brignell SC, Bron S, Brouillet S, Bruschi CV, Caldwell B, Capuano V et al (1997) The complete genome sequence of the gram-positive bacterium *Bacillus subtilis*. *Nature* **390:** 249-256

Ludwig H, Homuth G, Schmalisch M, Dyka FM, Hecker M, Stulke J (2001) Transcription of glycolytic genes and operons in *Bacillus subtilis*: evidence for the presence of multiple levels of control of the *gapA* operon. *MolMicrobiol* **41:** 409-422

McLachlan GJ, Krishnan T (1997) *The EM algorithm and Extensions*, New York: Wiley.

Milon P, Konevega AL, Gualerzi CO, Rodnina MV (2008) Kinetic checkpoint at a late step in translation initiation. *Mol Cell* **30:** 712-720

Milon P, Tischenko E, Tomsic J, Caserta E, Folkers G, La Teana A, Rodnina MV, Pon CL, Boelens R, Gualerzi CO (2006) The nucleotide-binding site of bacterial translation initiation factor 2 (IF2) as a metabolic sensor. *Proc Natl Acad Sci U S A* **103:** 13962-13967

Oh E, Becker AH, Sandikci A, Huber D, Chaba R, Gloge F, Nichols RJ, Typas A, Gross CA, Kramer G, Weissman JS, Bukau B (2011) Selective ribosome profiling reveals the cotranslational chaperone action of trigger factor in vivo. *Cell* **147:** 1295-1308

Piir K, Paier A, Liiv A, Tenson T, Maivali U (2011) Ribosome degradation in growing bacteria. *EMBO Rep* **12:** 458-462

Rocha EP, Danchin A, Viari A (1999) Translation in *Bacillus subtilis*: roles and trends of initiation and termination, insights from a genome analysis. *Nucleic Acids Res* **27:** 3567-3576

Schubert M, Lapouge K, Duss O, Oberstrass FC, Jelesarov I, Haas D, Allain FH (2007) Molecular basis of messenger RNA recognition by the specific bacterial repressing clamp RsmA/CsrA. *Nat Struct Mol Biol* **14:** 807-813

Steitz JA (1969) Polypeptide chain initiation: nucleotide sequences of the three ribosomal binding sites in bacteriophage R17 RNA. *Nature* **224:** 957-964

Tobisch S, Zuhlke D, Bernhardt J, Stulke J, Hecker M (1999) Role of CcpA in regulation of the central pathways of carbon catabolism in *Bacillus subtilis*. *JBacteriol* **181:** 6996-7004

Winter T, Winter J, Polak M, Kusch K, Mader U, Sietmann R, Ehlbeck J, van Hijum S, Weltmann KD, Hecker M, Kusch H (2011) Characterization of the global impact of low temperature gas plasma on vegetative microorganisms. *Proteomics* **11:** 3518-3530
