# Supplementary material for: Translation elicits a growth rate‐dependent, genome‐wide, differential protein production in Bacillus subtilis
Source: Mol Syst Biol. 2016 May 18;12(5):870. doi: 10.15252/msb.20156608 (PMC5683663; doi:10.15252/msb.20156608)
Supplement: Supplementary file 2 — Expanded View Figures PDF [file MSB-12-870-s002.pdf]

Expanded View Figures

Figure EV1. Molecular RNA species as function of the rate of growth.

A Total RNA abundance as function of the growth rate. Data are linearly fit (dashed line).
B First-order polynomial fit of total RNA abundance (black) and rRNA abundance (red, as calculated from the rRNA percentage within the total RNA pool; panels C and D). The 95% confidence intervals are shown with shaded areas.
C Percentage of each rRNA species (filled-in circles) in total RNA as function of the growth rate. rRNA sub-species are fit with a second-degree polynomial (dashed line). The corresponding 95% confidence band is given by the coloured area.
D Percentage of total rRNA species (filled-in circles) in total RNA as function of the growth rate obtained by summing every rRNA species (from panel C). Total rRNA is fit with a first-degree polynomial (dashed line). The corresponding 95% confidence band is given by the coloured area.
E Distribution histograms of one (out of four replicates) representative expression dataset (spike-in genome-wide array) per medium.
F Plots of a representative gene expression dataset from SE-, S-, CH- and CHG-grown cells vs. a representative gene expression dataset from CHG-grown cells. The red line corresponds to the bisector and the blue lines to linear fits. Blue and red lines are identical when plotting two replicates of the very same growth condition (CHG vs. CHG), while with increasing growth rates, expressions of an increasing number of genes are switched off.
G Normalized mRNA abundance of four constitutively expressed genes (see Fig 2B). The 95% confidence intervals are shown with bars. The corresponding datasets can be found in Dataset EV1.

▶

© 2016 The Authors

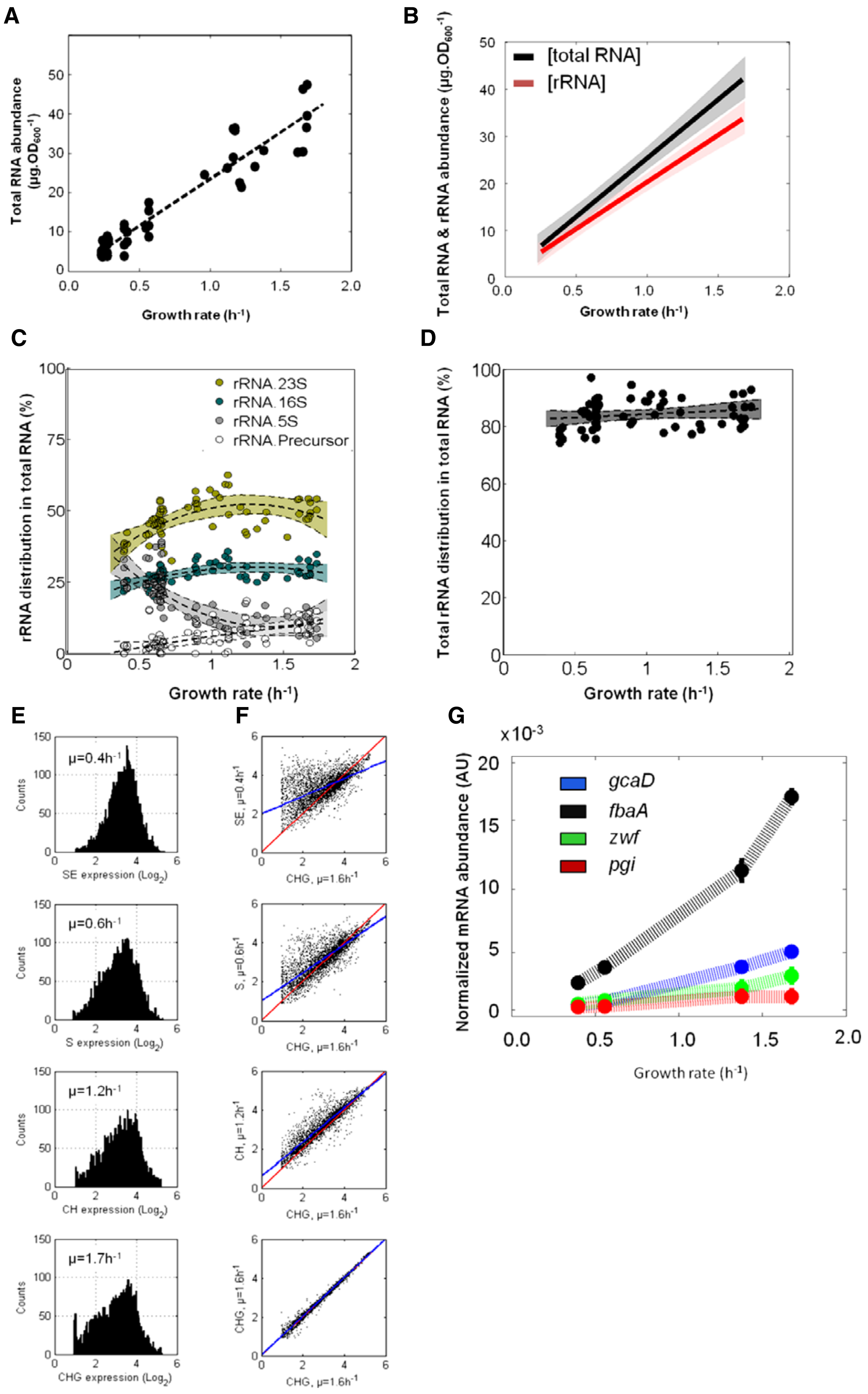

Figure EV1.

 © 2016 The Authors

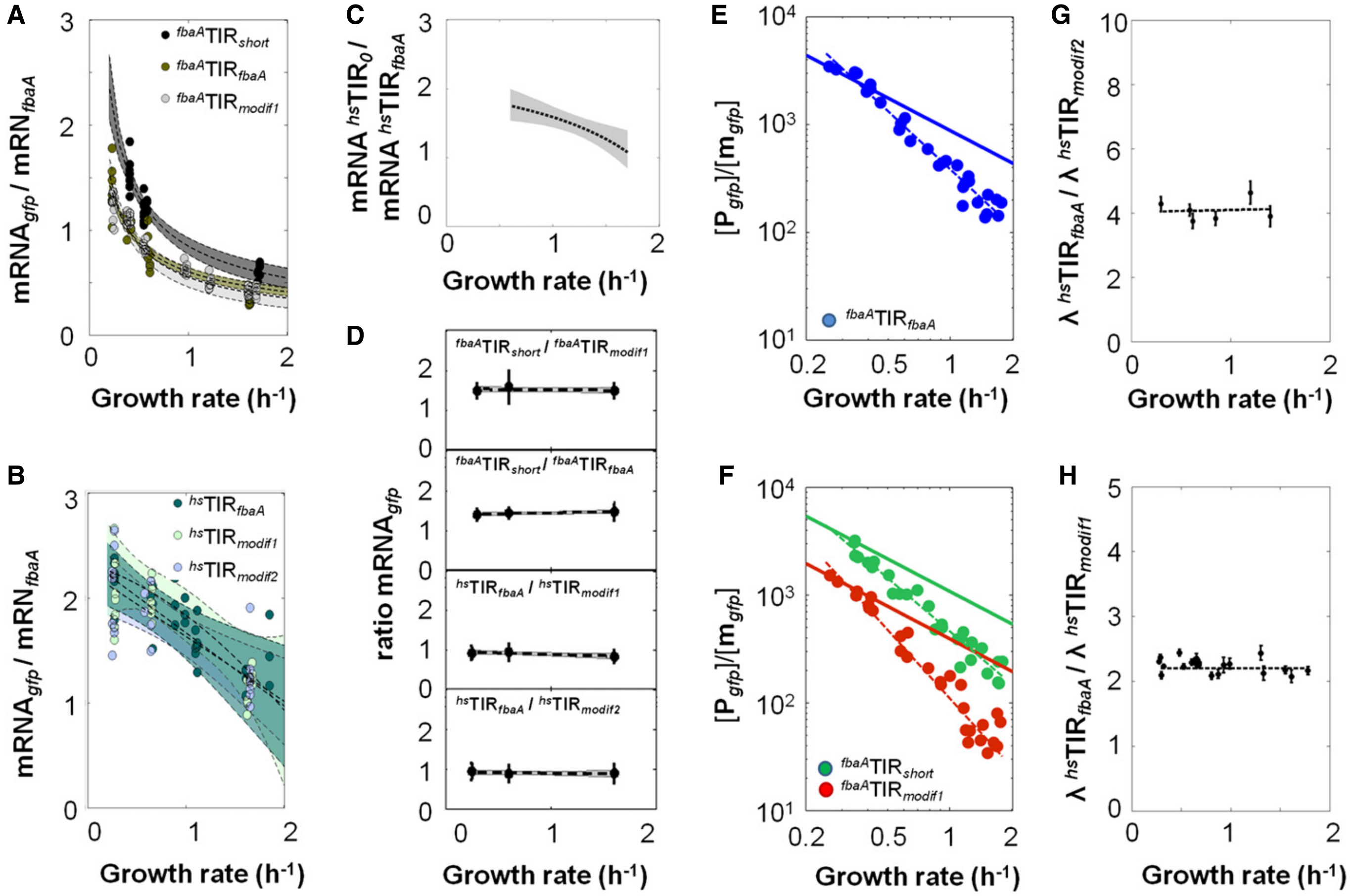

Figure EV2. Relative abundance of the *gfp* mRNA expressed under the control of the P_{fbaA} or P_{hs} promoters as function of the growth rate and the resulting ratios of translation efficiencies.

A, B Relative abundance (qPCR) of the *gfp* mRNA to the natural *fbaA* mRNA in the different strains as function of the growth rate. Filled-in circles correspond to 8 replicates per medium. Data are fit with a third-degree polynomial (dashed line) and the 95% confidence bands are given by the coloured area.

C The *gfp* mRNA produced by the ${}^{hs}TIR_0$ construct exhibits a differential stability with increasing growth rate as compared to that of ${}^{hs}TIR_{fbaA}$, the 95% confidence bounds is given as bar and the data are fit with a third-degree polynomial polynomial (dashed line).

D Ratios between the relative abundance of *gfp* mRNAs from the different strains and that of the ${}^{fbaA}TIR_{fbaA}$ strain are invariant with growth rate. Filled-in circles are the mean of the 8 replicates (from panel A) per medium; the 95% confidence bounds are given as bars and the data are fit with first-degree polynomial (dashed line).

E $\log_{10}\left(\frac{[P_{gfp}]}{[m_{gfp}]}\right)$ as a function of $\log_{10}(\mu)$ for the ${}^{fbaA}TIR_{fbaA}$ strain (in blue) using the dataset plotted on Fig 2 and linearly fitted (dashed line). The straight line has a slope of -1 and indicates a linear dependency to μ^{-1}.

F $\log_{10}\left(\frac{[P_{gfp}]}{[m_{gfp}]}\right)$ as a function of $\log_{10}(\mu)$ for the ${}^{fbaA}TIR_{modif1}$ and ${}^{fbaA}TIR_{short}$ (in red and green, respectively) using the dataset plotted in Fig 3 and linearly fitted (dashed lines). The straight line has a slope of -1 and indicates a linear dependency to μ^{-1}.

G, H The black dots represent ratios of translation efficiencies (λ) between two strains (${}^{hs}TIR_{fbaA}gfp$ vs. ${}^{hs}TIR_{modif2}gfp$ on panel G; ${}^{hs}TIR_{fbaA}gfp$ vs. ${}^{hs}TIR_{modif1}gfp$ on panel H) and 95% of confidence is given as vertical bars. The dashed curves are computed using the parameters estimated on Fig 4. The corresponding datasets can be found in Dataset EV2.

© 2016 The Authors

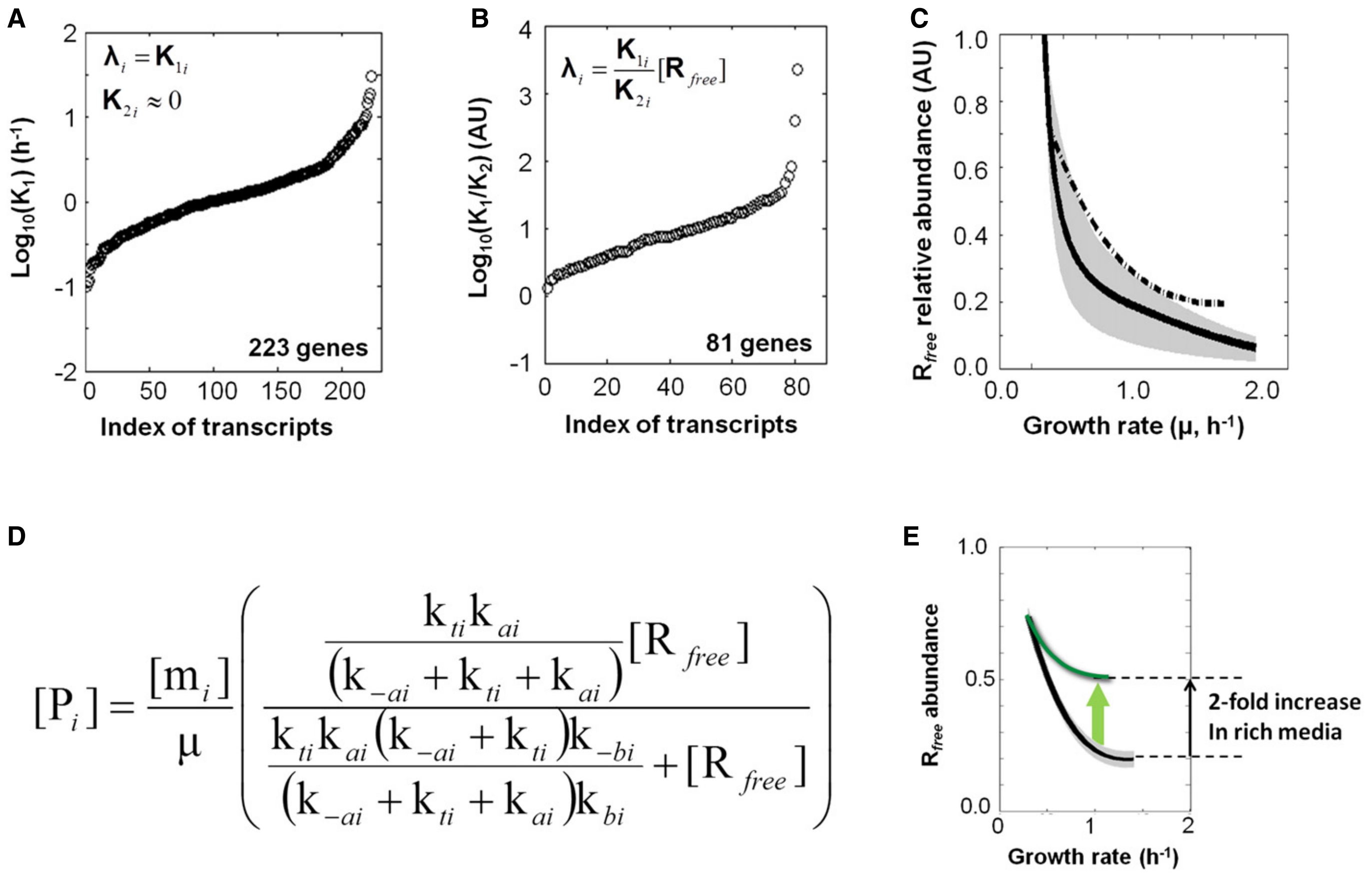

Figure EV3. Genome-wide estimation of the {K_{1i}, K_{2i}} pairs of each transcript and of *free* ribosome in *B. subtilis* using transcriptome and proteome datasets and a Michaelis–Menten-type rate law.

A Plot of the K_{1i} (in $\log_{10}$) of each of the 223 transcripts (*i*, index of transcripts) that exhibited a constant translation efficiency (R_{free}-saturated).

B Plot of the values of K_{1i}/K_{2i} ratio (in $\log_{10}$) of each of the 81 transcripts (*i*, index of transcripts) that exhibited a linear relationship between their translation efficiencies and R_{free} (R_{free}-undersaturated).

C Inference of R_{free} abundance from the genome-wide proteome and transcriptome datasets (black line); the 95% confidence interval is given by the grey area (see Appendix section 3.4). The *free* ribosome abundance at slow growth was assigned a value of 1.0 and *free* ribosome abundance in other conditions is reported normalized to this value. The *free* ribosome abundance estimated from the synthetic reporter library is represented with the dashed black line. The list of the transcripts corresponding to panels (A) and (B) can be found in Dataset EV3.

D Complete model of protein (P_i) production derived from Fig 7A, with the translation efficiency in parentheses (for more detail, see Appendix section 2).

E Putative R_{free} abundance variation with respect to the addition of a translation inhibitor. The R_{free} abundance in media with 1 mg ml^{-1} tetracycline (green line) compared with R_{free} abundance in media without tetracycline (black line).

Figure EV4. Graphical representation of the diversity of growth rate-dependent controls regulating protein production. ▶

A–C The ${}^{hs}TIR_{fbaA}$*gfp* transcript abundance increases with increasing growth rate (violet to grey), while the translation efficiency strongly decreases since the K_2 constant does not compensate for the drop in R_{free} abundance.

D–F The ${}^{fbaA}TIR_{short}$*gfp* transcript abundance increases with increasing growth rate (violet to grey), while the translation efficiency only slightly decreases because the K_2 constant partially compensates for the drop in R_{free} abundance.

G–I The ${}^{dhaS}TIR_{dhaS}$*gfp* transcript abundance decreases with increasing growth rate (violet to grey), which is the consequence of its chromosomal location at the vicinity of the terminus of replication. The translation efficiency strongly decreases since the K_2 constant does not compensate for the drop in R_{free} abundance.

J–L The ${}^{ackA}TIR_{ackA}$*gfp* transcript abundance strongly increases with increasing growth rate (violet to grey) and compensate for the strong drop in translation efficiency. The *ackA* gene is not supposed to be regulated neither positively nor negatively in the growth media used to reach the four different growth rates.

Data information: Plots showing GFP abundances (A, D, G and J) and GFP abundances (B, E, H and K) of the genetic constructs ${}^{hs}TIR_{fbaA}$*gfp*, ${}^{fbaA}TIR_{short}$*gfp*, ${}^{dhaS}TIR_{dhaS}$*gfp*, ${}^{ackA}TIR_{ackA}$*gfp*, respectively. Protein abundance (coloured area in C, F, I and L) at slow (violet) and fast (grey) growth depends on the dilution rate, the free ribosome abundance, the cognate transcript abundance and the Michaelis–Menten-type K_{2i} constant in growth- rate-dependent manner.

 © 2016 The Authors

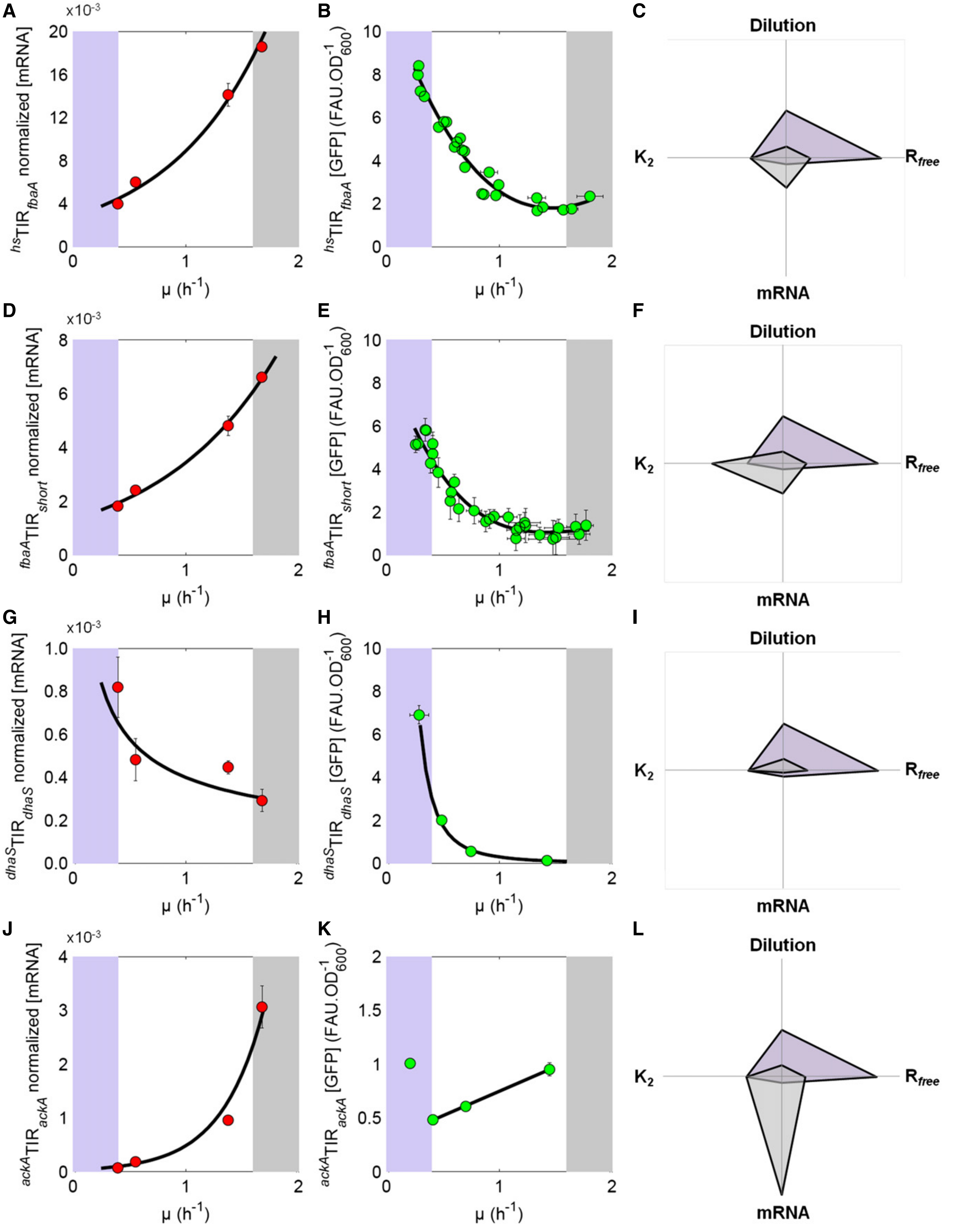
A
B
C
D
E
F
G
H
I
J
K
L
Dilution
K2
Rfree
mRNA
μ (h-1)
x10-3
hsTIRfbaA normalized [mRNA]
hsTIRfbaA [GFP] (FAU.OD600-1)
fbaATIRshort normalized [mRNA]
fbaATIRshort [GFP] (FAU.OD600-1)
dhaSTIRdhaS normalized [mRNA]
dhaSTIRdhaS [GFP] (FAU.OD600-1)
ackATIRackA normalized [mRNA]
ackATIRackA [GFP] (FAU.OD600-1)

Figure EV4.

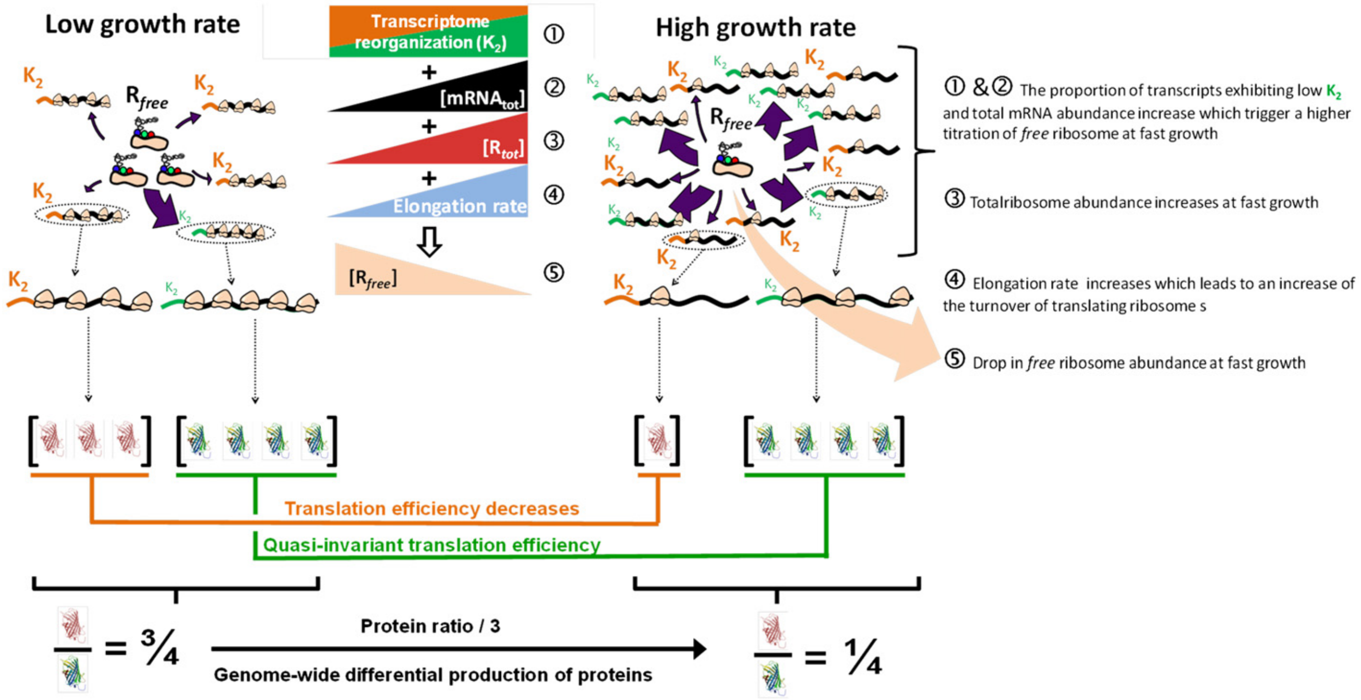

Figure EV5. Schematic representation of the interdependence of *free* ribosome abundance and both mRNA and ribosome abundances.
The K_2-related global reorganization of mRNA expression leads to a drop in R_{free} with increasing growth rate, which in turn promotes differential protein production. From low to high growth rates, the total mRNA and total ribosomal abundances as well as the protein elongation aggregated parameter (k_{pi}, Table 2; Bremer & Dennis, 2008) increase, while the *free* ribosome abundance drops. Titration of R_{free} is weaker at low than at fast growth because transcripts exhibiting strong K_1 and low K_2 (green TIR) are relatively upregulated and those exhibiting converse properties (orange TIR) downregulated with increasing growth rate. The ribosome density along the mRNA is the combined outcome of the translation efficiency and the elongation rate. The "red" and "green" proteins are differentially produced with increasing growth rate due to the growth rate-dependent translation efficiency.

 © 2016 The Authors
